# Physiological and Comparative Transcriptomic Analysis Provide Insight Into Cotton (*Gossypium hirsutum* L.) Root Senescence in Response

**DOI:** 10.3389/fpls.2021.748715

**Published:** 2021-10-18

**Authors:** Lingxiao Zhu, Liantao Liu, Hongchun Sun, Yongjiang Zhang, Jijie Zhu, Ke Zhang, Anchang Li, Zhiying Bai, Guiyan Wang, Cundong Li

**Affiliations:** ^1^State Key Laboratory of North China Crop Improvement and Regulation, Key Laboratory of Crop Growth Regulation of Hebei Province, College of Agronomy, Hebei Agricultural University, Baoding, China; ^2^Institute of Cereal and Oil Crops, Hebei Academy of Agricultural and Forestry Science, Shijiazhuang, China

**Keywords:** cotton, low nitrogen, root senescence, physiological, transcriptomic

## Abstract

Nitrogen (N) deficiency is one of the pivotal environmental factors that induce leaf senescence. However, little is known regarding the impact of low N on root senescence in cotton. Thus, the objective of this study was to investigate the effect of low nitrogen on root senescence. In this study, the molecular mechanism of cotton root senescence in response to nitrogen deficiency was investigated by combing physiological and transcriptomic analysis when no nitrogen and normal nitrogen (138mg N·kg^−1^ soil). The results showed that: (1) nitrogen starvation induced the premature senescence of leaf, while delaying root senescence. (2) The increase in catalase (CAT) activity at 60, 80, and 100days after emergence (DAE), combined with decrease of malonaldehyde content at 60, 80, and 100 DAE, and the content of abscisic acid (ABA), all of these contributed to the delay of root senescence by low nitrogen treatment. (3) To study the molecular mechanisms underlying root senescence, the gene expression profiling between low nitrogen and normal nitrogen treatments were compared pairwise at 20, 40, 60, 80, and 100 DAE. A total of 14,607 genes were identified to be differentially expressed at these five points. (5) Most genes involved in glutathione (GSH) and ascorbate peroxidase (APX) synthesis were upregulated, while ABA, apoptosis, caspase, and cell cycle-related differentially expressed genes (DEGs) were downregulated. Coupled with the physiology data, these results provide new insights into the effect of nitrogen starvation on root senescence.

## Introduction

Senescence represents the final stage of plant development and is characterized by nutrient redistribution from senescing organs to young organs (Urs and Fischer, 1994; Masclaux et al., 2000). Natural, age-dependent senescence is a well-orchestrated process that plays a vital role in plant development, and its initiation is dependent on the life cycle of the organism undergoing senescence (Lim et al., 2007; Woo et al., 2013). Cotton is one of the most important commercial crops in the world, contributing approximately 35% of the fiber used worldwide (Ma et al., 2018). However, early senescence in cotton plants, which is referred to as premature senescence, has appeared more frequently in cotton-growing countries due to various reasons (Dong et al., 2006; Dai and Dong, 2011), resulting in reduced lint yield and poor fiber properties (Wright, 1999). Therefore, clarifying the underlying mechanism of senescence of cotton will significantly improve cotton fiber quality and yields.

Senescence is a complex process influenced by the expression of thousands endogenous genes and various environmental factors (Noodén et al., 2010; Breeze et al., 2011), such as the light cycle (Wu et al., 2012), temperature (Li et al., 2021), water (Zhang et al., 2015), mineral nutrition (Yang et al., 2017), and pathomycete (Bancal et al., 2016). Phytohormones play pivotal roles in the regulation of senescence. Abscisic acid (ABA), jasmonic acid (JA), and ethylene (ETH) can promote senescence, whereas cytokinin can delay senescence (Ye, 2012; Jibran et al., 2013; Khan et al., 2013; Kong et al., 2013a; Feng et al., 2021). Senescence is usually accompanied by an increase in reactive oxygen species (ROS), such as superoxide and hydrogen peroxide, which leads to lipid peroxidation and cell damage (Trippi et al., 1989; De Simone and Johanningmeier, 1998). Meanwhile, maintaining the membrane intactness is a key issue in senescence regulation based on current information (Stefan and Urs, 2002). Cotton senescence is a process of gradual deterioration that terminates growth and eventually results in plant death. Chen and Dong (2016) proposed that source-sink coordination, the relationship between vegetative and reproductive growth, root–shoot relation, and the balance of carbon and nitrogen (N) metabolism are the crucial mechanisms involved in cotton senescence.

For decades, senescence has been exhaustively studied in leaves. Leaf senescence is usually evaluated by leaf yellowing or a decrease in chlorophyll content (Levey and Wingler, 2005). However, leaf senescence is far more complicated than the color change due to chlorophyll loss and chloroplast disassembly (Astrid et al., 2006; Zhao et al., 2012; Tang et al., 2015). Leaf senescence is a developmental process that involves the degradation of macromolecules such as nucleic acids, proteins, and ribulose-1,5-bisphosphate carboxylase, together with the accumulation of ROS and increases in proteinase activities, lipid peroxidation, and membrane leakiness (Vicky, 1997; Río et al., 1998; Saeid et al., 2003; Assaf et al., 2014). Leaf senescence can dramatically affect crop production (Gregersen et al., 2010). In severe cases, the leaves are small and thin, with yellowing extending throughout the whole leaves along the veins (Ma et al., 2014).

However, although roots play pivotal roles in plant development, including in the main organs’ uptake of water and nutrients, as well as the biosynthesis and transport of hormones such as ABA and cytokinins (Rachmilevitch et al., 2006; Ehdaie et al., 2010), less research has focused on roots than on the leaves of plants because of their limited accessibility in the soil. The root system can regulate leaf senescence directly or indirectly through the biosynthesis and transport of cytokinins and ABA (Dong et al., 2008). A strong negative correlation was observed between the extent of plant senescence and root biomass in wheat (Hebbar et al., 2014). In *Alhagi sparsifolia*, the inhibition of root respiration accelerated leaf senescence (Tang et al., 2019). In wheat, a deep root system is pivotal in regulating plant senescence (Kong et al., 2013b). Fanello et al. (2019) suggested that roots may not undergo a clear-cut senescence process as leaves do. Moreover, it is currently not clear whether sustained root growth is the cause or the consequence of delayed leaf senescence (Kamh et al., 2005).

As a major macronutrient and limiting factor for agricultural crops, N has a profound influence on plant growth and development, and nitrogen deficiency induces crop senescence (Hirel et al., 2007; Tschoep et al., 2010). Nitrogen stress is one of the dominant causes of leaf senescence in wheat (Criado et al., 2007). In *Arabidopsis*, Park et al. (2019) proved that *ORE1* is the key factor that induces leaf senescence under nitrate starvation. In winter oilseed rape, transcription factors (TFs) of the *AP2/ERF*, *WRKY*, *NAC*, and *MYB* families are involved in nitrogen deficiency-related regulatory networks (Koeslin-Findeklee et al., 2015). Nitrogen deficiency results in poor cotton plant development and premature senescence (Zhang et al., 2011). Increasing soil nitrogen content can delay cotton leaf senescence (Dong et al., 2012). Moreover, Si and H_2_S can delay leaf senescence through alleviating damage by regulating the expression of senescence associated genes (SAGs) under nitrogen starvation (Cylia et al., 2018; Zhang et al., 2020).

To date, although several mechanisms involved in leaf senescence have been identified under nitrogen stress in some plants, there is still little published information about the regulatory mechanism of nitrogen during root senescence in cotton at the physiological level and through transcriptomics. To address this knowledge gap, we conducted a transcriptomics study with the aim of explaining the molecular mechanisms through which nitrogen affects root senescence. Therefore, the objectives of this study were to: (i) examine the effects of nitrogen deficiency on root senescence; (ii) determine whether root senescence is consistent with leaf senescence; and (iii) characterize the physiological and transcriptomics response of root senescence under nitrogen starvation.

### Materials and Methods

### Plant Material, Growth Conditions, and Nitrogen Treatments

The experiment was conducted during August to November of 2020 in a smart greenhouse at Hebei Agricultural University (38°85'N, 115°30'E) in Baoding Hebei Province, China. A commercial transgenic *Bacillus thuringiensis* cotton (*Gossypium hirsutum* L.) cultivar, Guoxin9, developed by the Guoxin Rural Technical Service Association was used in this experiment.

The seeds were surface-sterilized with a solution of 5% (v/v) NaClO for 10min followed by five washes with sterile distilled water and then germination in an incubator at 25°C for 24h without light. Seeds that consistently germinated were transplanted into polyvinylchloride (PVC) columns (55cm in height and 20cm in diameter; one plant per PVC column) containing a 16-kg mixture of topsoil. Infertile sandy loam was selected after being air-dried and passed through a 20-mesh sieve as a culture medium. The loam was collected at a depth of 10–30cm to ensure N deficiency along the fence line of a long-term experimental site in Baoding, Hebei, China (38°85'N, 115°30'E). It contained 0.86g·kg^−1^ organic matter, 13.23mg·kg^−1^ alkali-hydrolyzable N, 3.94mg·kg^−1^ available P, and 100.42mg·kg^−1^ available K. There were two levels of nitrogen: low nitrogen, at 0g N·kg^−1^ soil; and normal nitrogen, at 138mg N·kg^−1^ soil (Coviella et al., 2002), in which urea was used as the nitrogen fertilizer. Next, 70mg P·kg^−1^ soil (Ye, 2012) and 14mg K·kg^−1^ soil (Read et al., 2006) were applied to each PVC column, using superphosphate [Ca(H_2_PO_4_)_2_·H_2_O] and potassium chloride (KCl) as sources of phosphorus and potassium, respectively. After emergence, 500ml of a solution of supplemental micronutrients and iron salts was added to each PVC column per week, which included (mM) 2.5 CaCl_2_, 1 MgSO_4_, 2×10^−4^ CuSO_4_, 1×10^−3^ZnSO_4_, 0.1 Fe Na EDTA, 2×10^−3^ H_3_BO_3_, 5×10^−6^ H_2_MoO_4_, and 1×10^−3^ MnSO_4_ (Ye, 2012). The plant was grown in a smart greenhouse under day/night temperatures of 28/25°C and a 14/10h (light/dark) photoperiod, a relative humidity of 45–50%, and a light intensity of 600μmol·m^−2^·s^−1^ during the day. For the physiological and gene expression analyses of the roots, samples were obtained by washing the roots (remove the taproot) with running water in a 20-mesh nylon mesh bag and then frozen immediately in liquid nitrogen at 20, 40, 60, 80, and 100days after emergence (DAE), and then stored at −80°C.

### Measurement of Aboveground Traits

From 20 DAE, the net photosynthetic rate (Pn) and soil and plant analyzer development (SPAD) value of the cotton were determined every 20days until harvest (at 20, 40, 60, 80, and 100 DAE). The relative chlorophyll content of the top four functional leaves were determined using a SPAD meter (SPAD-502, Konica-Minolta, Tokyo, Japan) with eight replicates. The Pn of the top four leaves was measured using a portable photosynthesis system (LI-6400, Li-COR, Lincoln, NE, United States) from 9:00 to 11:00 with eight replicates. The main parameters of the photosynthesis system were established as follows: irradiation=600μmol·m^−2^·s^−1^, air flow=500μmol·s^−1^, and CO_2_ concentration=400μmol·mol^−1^.

### Measurement of Root Morphological Traits and Nitrogen Content

After harvest, the roots and aboveground part of the plant were separated at the cotyledonary node. The root system was obtained by washing the roots with running water in a 20-mesh nylon mesh bag. The roots were placed separately in a transparent polyvinyl chloride box containing 2mm of water. The roots were then scanned using an EPSON-V700 scanner (Epson-V700, Suwa, Japan) at 600 dpi, and then by means of the software WinRHIZO REG2009 (Regent Instruments, Inc., QC, Canada) to obtain the root length, root surface area, root diameter, and root volume.

The harvested leaves and roots samples from each treatment were exposed to 105°C for 30min, and then dried at 80°C to a constant weight, and then ground into powder for the determination of total N content. The total N was determined by the semi-trace kjeldahl method using a continuous flow analyze (AA3, SEAL, Germany).

### Measurement of Root Physiological Traits

The activity of superoxide dismutase (SOD), peroxidase (POD), catalase (CAT), and the content of soluble sugar, soluble protein, and malonaldehyde (MDA) were determined with the corresponding assay kits (Jiancheng Biotechnology, Nanjing, China).

Phytohormones, indole-3-acetic acid (IAA), gibberellin A3 (GA3), zeatin riboside (ZR), methyl jasmonate (JA-ME), and ABA in roots were extracted and purified according to the method described in Wu et al. (2019). About 0.3g of fresh roots was homogenized in 2ml 80% methanol and stored at 20°C for 48h. The extract was centrifuged at 4,000*g* for 15min at 4°C, and then the supernatant was passed through C18 Sep-Pak cartridges (Waters Corp., Millford, MA, United States). The sediments were re-suspended with 10ml of 100% (v/v) methanol and 10ml of ether. Afterward, the eluate was dried using pure N_2_ at 20°C, and then stored at 40°C. Endogenous free IAA, GA3, ZR, JA-ME, and ABA contents were calculated according to the description by Weiler et al. (1981).

### RNA Extraction, Sequencing, and Data Processing

Total RNA of each sample was extracted using the RNeasy Plant Mini kit (Qiagen, Valencia, CA, United States). RNA degradation and contamination were monitored on 1% agarose gels. RNA purity was checked using the NanoPhotometer® spectrophotometer (IMPLEN, CA, United States). RNA integrity was assessed using the RNA Nano 6000 Assay Kit of the Bioanalyzer 2100 system (Agilent Technologies, CA, United States). The library was constructed on an Illumina HiSeq 6000 platform, and a total of 30 transcriptome libraries were constructed. Raw data (raw reads) of the fastq format were firstly processed through in-house perl scripts. In this step, clean data (clean reads) were obtained by removing reads containing adapters, reads containing Ploy-N, and low quality reads from raw data. After removing the low mass readings, 201.15GB of clean data were obtained (Supplementary Table S1). At the same time, the Q20, Q30, and GC contents of the clean data were calculated. The percentage of Q20 and Q30 values of all clean reads exceeded 98 and 94%, respectively. The error rate was less than 0.02%. All the downstream analyses were based on the high quality clean data.

The reference genome and gene model annotation files were downloaded from the genome website directly. The statistics of the comparison between reads and the conference genome were shown in Supplementary Table S2. The index of the reference genome was built using Hisat2 v2.0.5 and paired-end clean reads were aligned to the reference genome using Hisat2 v2.0.5. We selected Hisat2 as the mapping tool because Hisat2 can generate a database of splice junctions based on the gene model annotation file and thus produce a better mapping result than other non-splice mapping tools. FeatureCounts v1.5.0-p3 was used to count the read numbers mapped to each gene. Then the fragments per kilobase of transcript sequence per million base pairs sequenced (FPKM) of each gene was calculated based on the length of the gene and the read count mapped to this gene. FPKM considers the effect of sequencing depth and gene length for the read count at the same time, and is currently the most commonly used method for estimating gene expression levels.

Differential expression analysis between the low nitrogen treatment and the normal nitrogen treatment was performed using the DESeq2 R package (1.16.1). DESeq2 provides statistical routines for determining differential expression in digital gene expression data using a model based on the negative binomial distribution. The resulting *p-values* were adjusted using Benjamini and Hochberg’s approach for controlling the false discovery rate. Genes with an adjusted *value* of *p*<0.05 found by DESeq2 were assigned as differentially expressed genes (DEGs). Prior to differential gene expression analysis, for each sequenced library, the read counts were adjusted by the edgeR program package through one scaling normalized factor. Differential expression analysis of two conditions was performed using the edgeR R package (3.18.1). The *p-values* were adjusted using the Benjamini and Hochberg method. A corrected *p-value* of 0.05 (value of *p*<0.05) and absolute fold change of 2 (≥1) were set as the threshold for significantly differential expression.

Gene ontology (GO) enrichment analysis of DEGs was implemented by the clusterProfiler R package, in which the gene length bias was corrected. GO terms with a corrected *p-value* less than 0.05 were considered significantly enriched by differentially expressed genes. The Kyoto Encyclopedia of Genes and Genomes (KEGG) is a database resource for understanding high-level functions and utilities of biological systems, such as the cell, the organism, and the ecosystem, from molecular-level information, especially large-scale molecular datasets generated by genome sequencing and other high-throughput experimental technologies.^1^ We used the clusterProfiler R package to test the statistical enrichment of differentially expressed genes in KEGG pathways.

A weighted gene co-expression network analysis (WGCNA) was executed using R software on differentially expressed transcripts. The WGCNA algorithm is based on the assumed gene network without scale distribution. It then constructs a gene co-expression correlation matrix and gene network, defines them as adjacency functions, and analyzes and calculates the dissimilarity coefficients of different nodes to construct a hierarchical clustering tree (Chen et al., 2020a). Cytoscape_3.7.1 was used to draw the weighted gene co-expression network graph. Each node in the network represents a gene, and the edges represent the regulatory relationship between genes.

### qRT-PCR Validation

To determine the reliability of transcriptomic results, we selected 10 candidate DEGs that were highly related to nitrogen stress for qRT-PCR. The same total RNA that had been isolated from the roots of three biological replicates was used for qPCR. The yield of RNA was determined using a NanoDrop 2000 spectrophotometer (Thermo Scientific, United States), and the integrity was evaluated using agarose gel electrophoresis stained with ethidium bromide. Quantification was performed under following parameter on LightCycler® 480 II Real-time PCR Instrument (Roche, Swiss): in the first cycle of 95°C for 5min, in the second cycle, of 95°C for 10s, and 60°C for 30s. All primer sequences were designed using Generay Biotech (Generay, PRC; Supplementary Table S3).

### Data Processing and Statistical Analysis

All of the statistical analyses were performed using SPSS Statistics 26.0 (IBM, Armonk, NY, United States). Data are presented as means±SE values. A one-way ANOVA was conducted, and a Student’s *t*-test was used to compare treatment means at the 5% level.

## Results

### Responses of Aboveground Traits to Nitrogen Stress

**Figure 1:**
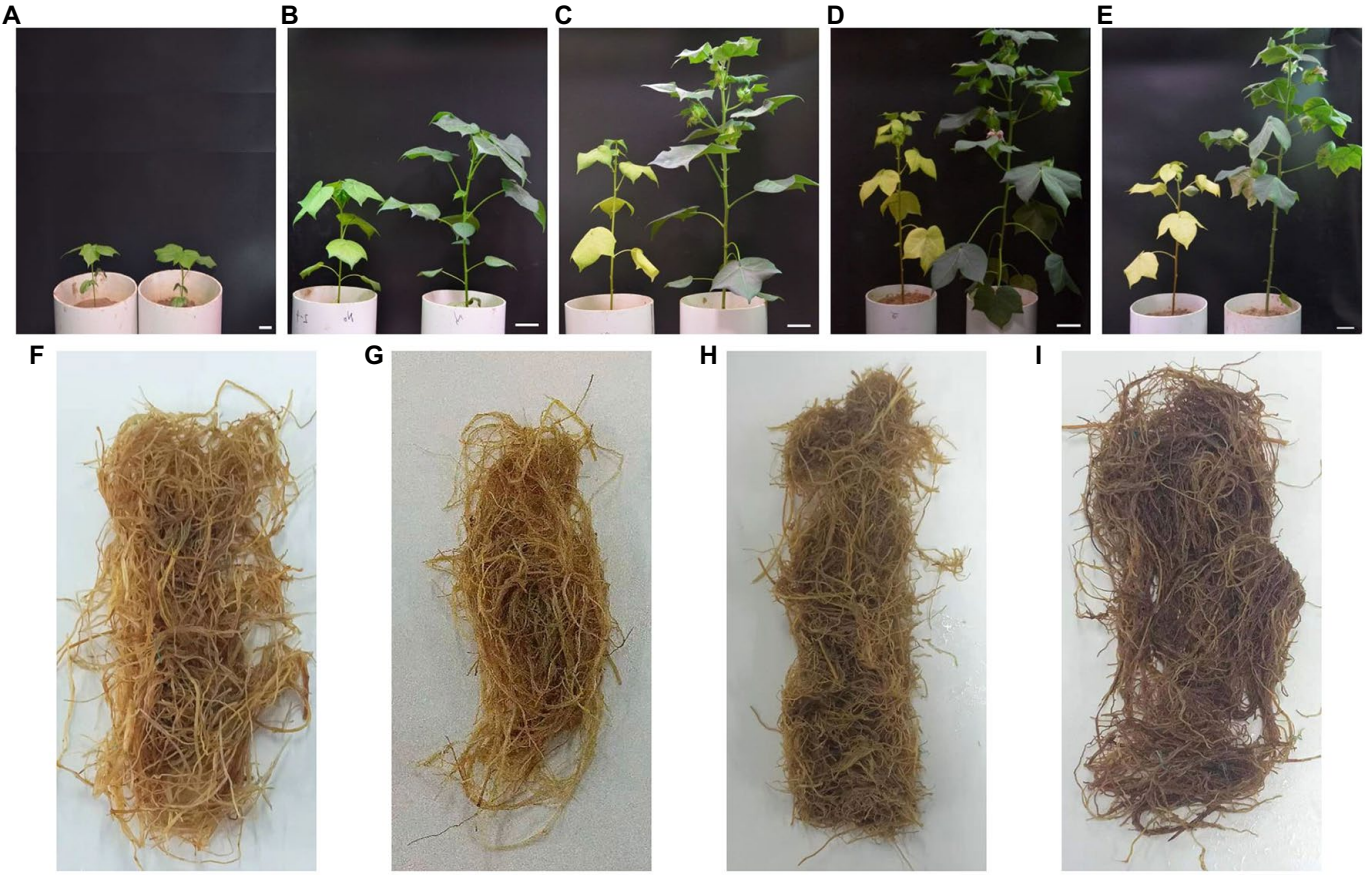
Photographs of dynamic changes in cotton plants and roots under different nitrogen (N) treatments. **(A–E)** represent the cotton plant at 20, 40, 60, 80, and 100days after emergence (DAE), respectively. **(F,G)** Represent the cotton root at 80 and 100DAE under low N treatment; **(H,I)** represent the cotton root at 80 and 100days after emergence under normal nitrogen treatment. For each picture of **(A-E)**, the left side is the low nitrogen treatment, and the right side is normal nitrogen treatment. Scale bar **(A-E)**=2cm.

Nitrogen starvation significantly inhibited the growth of shoots (Figure 1). The Pn and SPAD value under low nitrogen treatment significantly decreased after 20 DAE as the leaves etiolated (Supplementary Figure S1). The low nitrogen treatment reduced the Pn and SPAD value by 26.77 and 41.29% at 40 DAE, respectively, compared with the normal nitrogen treatment. At 100 DAE, the Pn and SPAD value of the low nitrogen treatment were only 52.42 and 36.45% of normal nitrogen treatment, respectively. These results indicated that nitrogen starvation induced the premature senescence of the aboveground part of cotton plants.

### Morphological Responses of Roots to Nitrogen Stress

Roots under low nitrogen treatment showed significantly higher morphological parameters except diameter than those for normal nitrogen treatment at 20 DAE (Supplementary Figure S2). However, all morphological parameters under low nitrogen treatment were markedly lower than those values under normal nitrogen treatment after 20 DAE. The total root length, surface area, and volume under normal nitrogen treatment were twice as large as those under low nitrogen treatment. The dynamic change in root diameter was basically the same under different nitrogen treatments. The low nitrogen treatment significantly decreased root diameter by 3.22 and 3.38% at 40 and 100 DAE, respectively, compared with the normal nitrogen treatment. These results suggested that nitrogen stress promoted root growth at the early stage, whereas inhibited root growth with the extension of stress time.

### Physiological Responses of Roots to Nitrogen Stress

The low nitrogen treatment significantly decreased the soluble protein content, which remained low throughout the growth period, compared with the normal nitrogen treatment (Supplementary Figure S3). The soluble protein content under the low nitrogen treatment ranged from 1.31 to 4.30mg/g, whereas it ranged from 4.12 to 10.26mg/g under the normal nitrogen treatment. Similarly, the low nitrogen treatment significantly decreased soluble sugar content. However, the dynamic change in soluble sugar content was basically the same under different nitrogen treatments.

**Figure 2:**
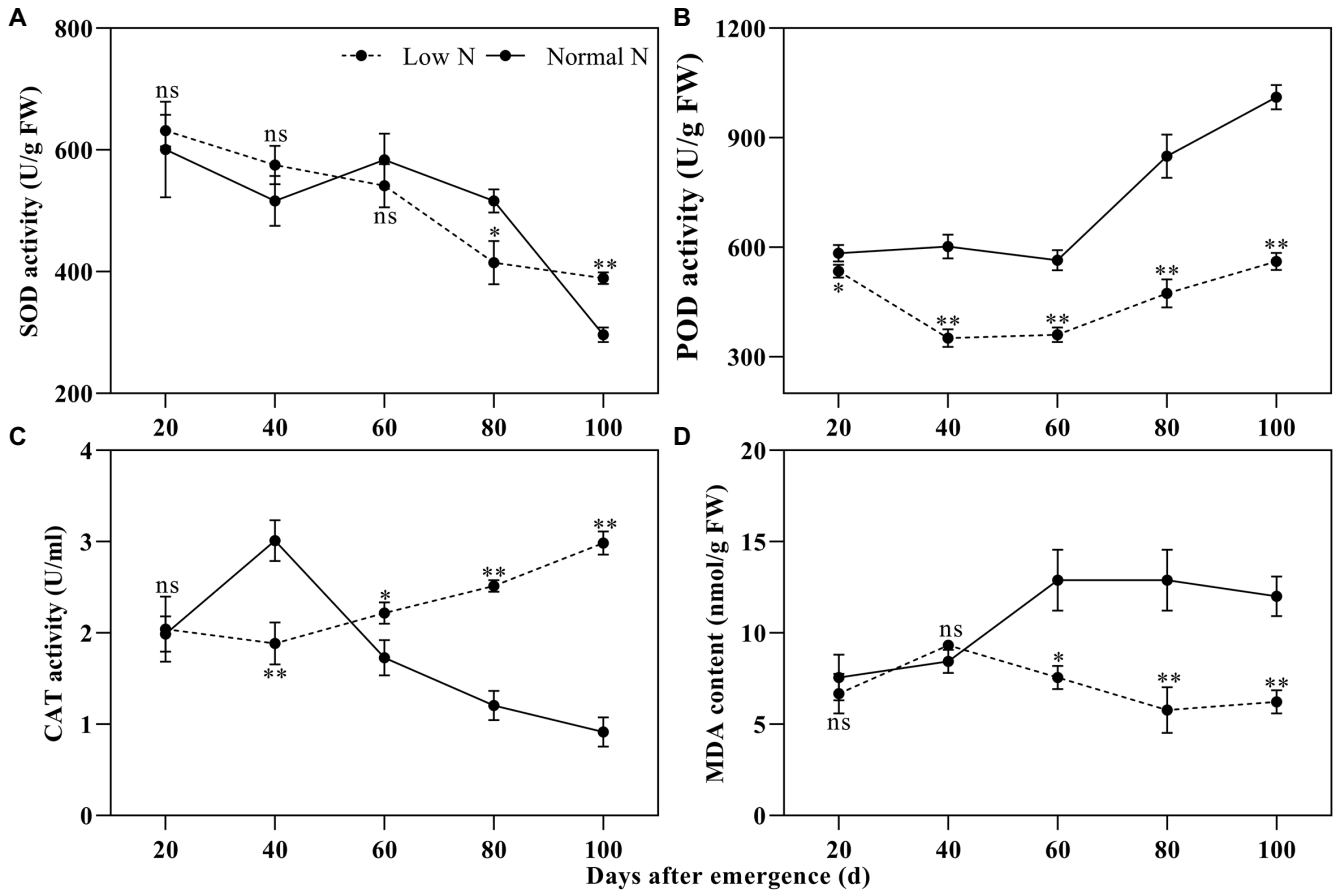
Dynamic changes in the superoxide dismutase (SOD) activity **(A)**, peroxidase (POD) activity **(B)**, catalase (CAT) activity **(C)**, and malondialdehyde (MDA) content **(D)** of cotton roots under different nitrogen treatments. Depicted are the means of three replicates±SE. ns, not significant (*p*>0.05), ^*^p<0.05, ^**^p<0.01.

Peroxidase activity differed significantly between the two nitrogen treatments, while there was no difference in SOD activity between the two nitrogen treatments until 80 DAE (Figures 2A,D). POD activity under the low nitrogen treatment was significantly lower than that under the normal nitrogen treatment. Compared with the normal nitrogen treatment, the SOD activity under the low nitrogen treatment was reduced by 19.67% at 80 DAE, whereas it increased by 31.42% at 100 DAE. Like the SOD activity, the low nitrogen treatment decreased CAT activity at 40 DAE, but increased CAT activity at 60, 80, and 100 DAE (Figure 2C). There were no statistical differences in MDA content between the two nitrogen treatments until 60 DAE (Figure 2D). At 60, 80, and 100 DAE, low nitrogen treatment markedly reduced the MDA content by 41.35, 55.16, and 48.17%, respectively, compared with the normal nitrogen treatment.

**Figure 3:**
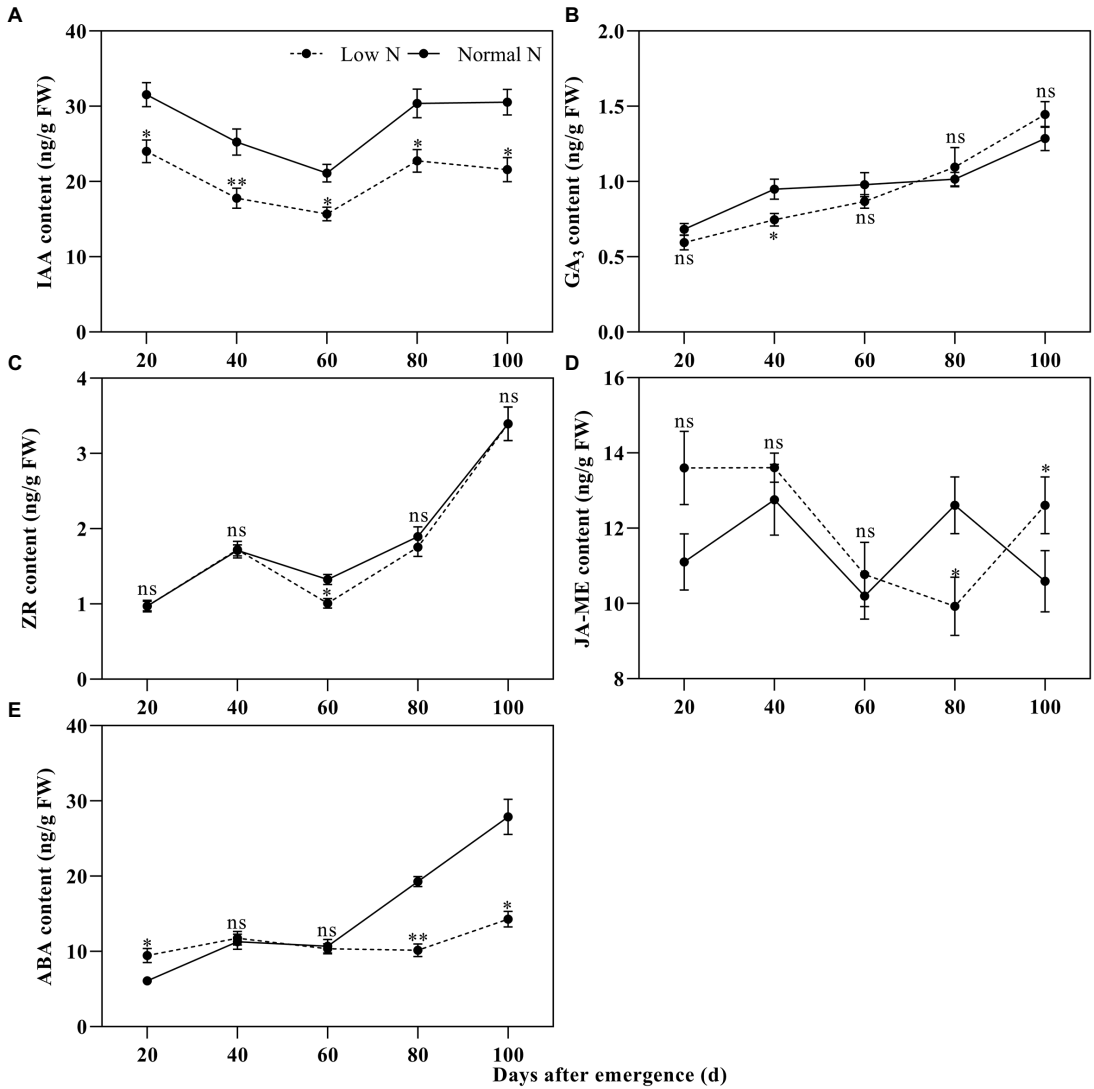
Dynamic changes in the indole-3-acetic acid (IAA; **A**), gibberellin A3 (GA_3_; **B**), zeatin riboside (ZR; **C**), methyl jasmonate (JA-ME; **D**), and abscisic acid (ABA; **E**) content of cotton roots under different nitrogen treatments. Depicted are the means of three replicates±SE. ns, not significant (*p*>0.05), ^*^p<0.05, ^**^p<0.01.

Compared with the normal nitrogen treatment, at 20, 40, 60, 80, and 100 DAE, low nitrogen treatment significantly reduced the IAA content of roots by 23.87, 29.58, 25.63, 25.14, and 29.38%, respectively (Figure 3). There were no statistically significant differences in GA_3_ content, except for at 40 DAE, and there were no statistically significant differences in ZR content, except for at 60 DAE. There was no difference in JA-ME content between the two nitrogen treatments until 80 DAE. JA-ME content under low nitrogen treatment was decreased by 19.67% at 80 DAE, whereas it increased by 19.07% at 100 DAE, compared with normal nitrogen treatment. ABA content under the low nitrogen treatment was increased by 55.68% at 20 DAE, whereas it decreased by 47.31% at 80 DAE and 48.82% at 100 DAE, compared with normal nitrogen treatment. Meanwhile, we found that the root color under normal nitrogen treatment was darker than that under low nitrogen treatment (Figure 1).

**Figure 4:**
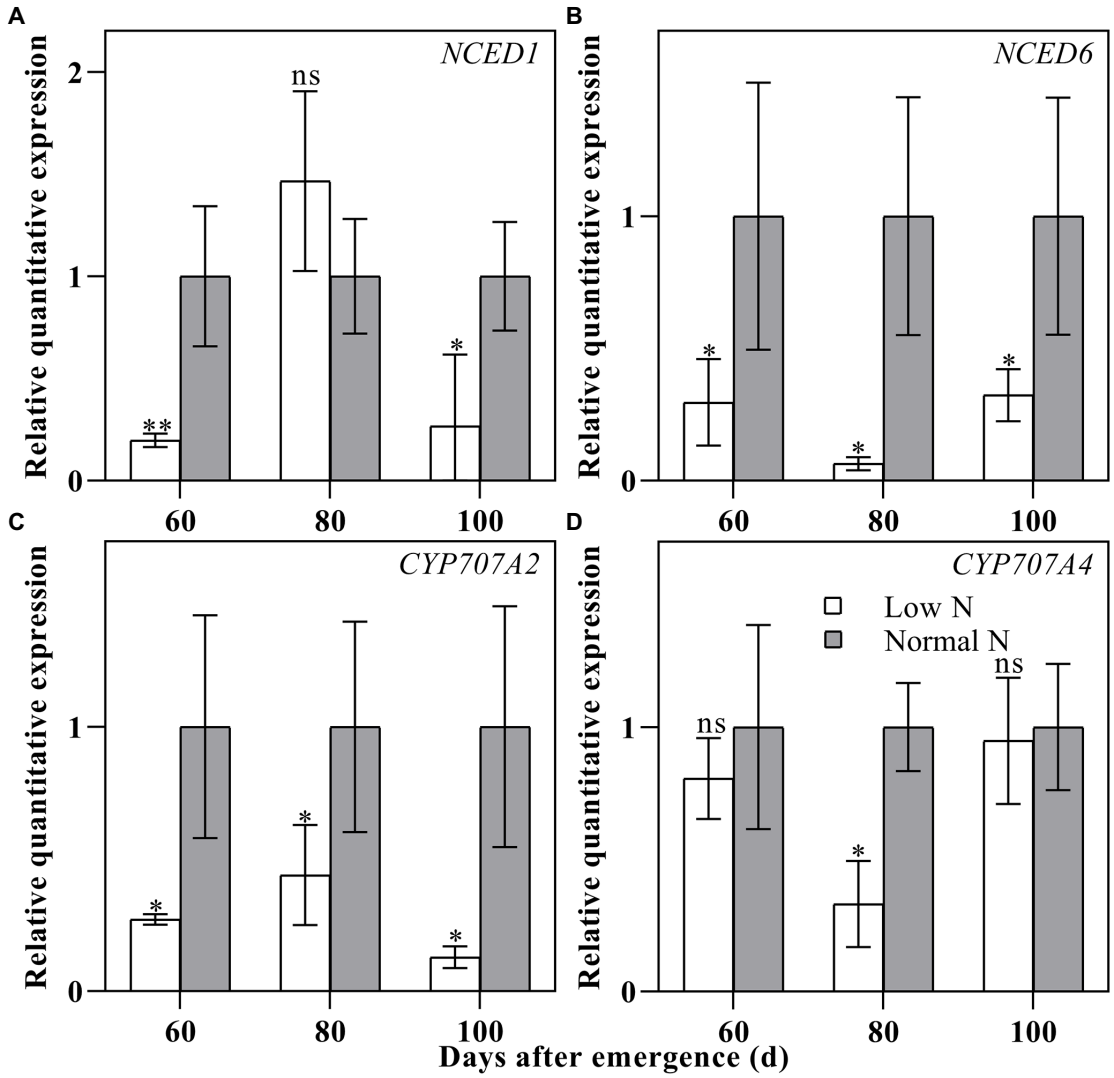
Relative quantitative expressions of *NCED1* **(A)**, *NCED6* **(B)**, *CYP707A2* **(C)**, and *CYP707A4* **(D)** in cotton roots under different nitrogen treatments. Depicted are the means of three replicates±SE. ns, not significant (*p*>0.05), ^*^p<0.05, ^**^p<0.01.

To verify the reliability of ABA content, the relative quantitative expressions of two ABA synthesis genes and two ABA catabolic genes were measured. The results showed that the relative quantitative expressions of ABA synthesis genes *NCED1* and *NCED6* and ABA catabolic genes *CYP707A2* and *CYP707A4* for low nitrogen treatment were downregulated except for at 80 DAE of *NCED1* (Figure 4). This was basically consistent with the results of ABA content analysis.

Compared with the normal nitrogen treatment, low nitrogen treatment significantly reduced the nitrogen content of leaves and roots (Supplementary Figure S4). The nitrogen content of leaves under the low nitrogen treatment at 20, 40, 60, 80, and 100 DAE was 30.86, 37.62, 29.80, 32.90, and 28.46% of that under normal nitrogen treatment, respectively. Similarly, the nitrogen content of roots under the low nitrogen treatment at 20, 40, 60, 80, and 100 DAE was 36.34, 52.65, 47.16, 72.51, and 56.21% of that under normal nitrogen treatment, respectively. Compared with normal nitrogen treatment, low nitrogen treatment reduced the nitrogen content in leaves and promotes leaf senescence, while reduced the nitrogen content in roots but delaied root senescence, indicating that nitrogen content was a negative regulator of leaf senescence, but a positive regulator of root senescence.

### Identification of Differentially Expressed Genes

**Figure 5:**
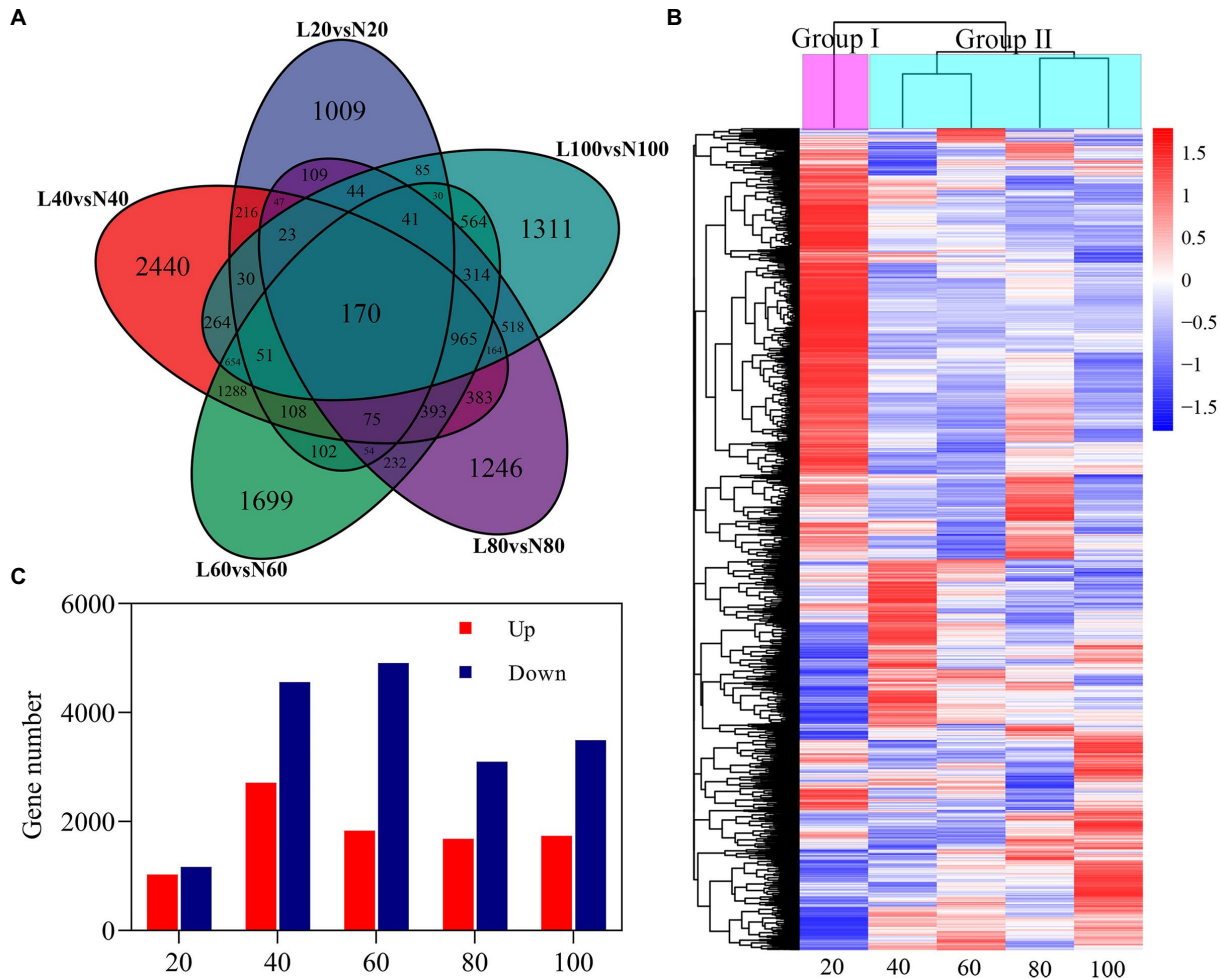
Differentially expressed genes (DEGs) in roots in response to nitrogen stress. **(A)** Venn diagrams of five comparisons (20, Low N 20 vs. Normal N 20; 40, Low N 40 vs. Normal N 40; 60, Low N 60 vs. Normal N 60; 80, Low N 80 vs. Normal N 80; and 100, Low N 100 vs. Normal N 100). DEGs at different stages, **(B)** up- and downregulated genes of five comparisons at different stages, and **(C)** hierarchy clustering of five comparisons of DEGs. The rows in the heat map represent genes, and the columns indicate comparisons between low nitrogen and normal nitrogen. The colors in the heat map indicate the values of log_2_ fold changes.

Differentially expressed genes were selected according to a threshold of |log_2_ fold change|≥1 with value of *p* (adjusted)≤0.05 between low nitrogen and normal nitrogen treatment. Compared with the nitrogen treatment, a total of 14,607 genes were identified to be differentially expressed (Figure 5). There were 2,194 (1,027 upregulated/1,169 downregulated), 7,271 (2,714 upregulated/4,557 downregulated), 6,740 (1,833 upregulated/4,907 downregulated), 4,778 (1,680 upregulated/3,098 downregulated), and 5,228 (1,735 upregulated/3,493 downregulated) DEGs at 20, 40, 60, 80, and 100 DAE, respectively. The highest numbers of DEGs was found at 40 DAE. The number of DEGs expressed only at 20, 40, 60, 80, and 100 DAE was 1,009, 2,440, 1,699, 1,246, and 1,311, respectively. There were 170 DEGs expressed at all five time points. These DEGs were clustered into two groups according to their log_2_ fold change values. Group I was primarily the vegetative growth stage, and Group II was the reproductive growth stage.

### Analyses of Growth-Stage-Specific DEGs

There were 1,009 DEGs only expressed in Group I, with 463 genes upregulated and 546 genes downregulated. For the upregulated genes, GO terms were mainly enriched in drug transmembrane transport (GO: 0006855, *p*<0.01), drug transport (GO: 0015893, *p*<0.01), and response to drug (GO: 0006855, *p*<0.01) in the biological process (BP) categories; apoplast (GO: 0048046, *p*<0.001), and extracellular region (GO: 0005576, *p*<0.01) in the cellular component (CC) categories; and transferase activity (GO: 0016747, *p*<0.001) and ATPase activity (GO: 001687, *p*<0.01) in the molecular function (MF) categories (Supplementary Figure S5A). For the downregulated genes, GO terms were mainly enriched in small molecule catabolic process (GO: 0044282, *p*<0.001) in the BP categories; photosynthetic membrane (GO: 0034357, *p*<0.0001), thylakoid (GO: 0009579, *p*<0.0001), and thylakoid part (GO: 0044436, *p*<0.0001) in the CC categories; and acyl-CoA dehydrogenase activity (GO: 0003995, *p*<0.0001), oxidoreductase activity (GO: 0016627, *p*<0.001), flavin adenine dinucleotide binding (GO: 0050660, *p*<0.01), and phosphoric ester hydrolase activity (GO: 0042578, *p*<0.01) in the MF categories (Supplementary Figure S5C). KEGG enrichment analysis showed that the pathways of upregulated genes were mapped to ether lipid metabolism (ath00565, *p*<0.01), glycerophospholipid metabolism (ath00564, *p*<0.05), ascorbate and aldarate metabolism (ath00053, *p*<0.05), and galactose metabolism (ath00052, *p*<0.05; Supplementary Figure S5B). The downregulated genes were mapped to valine, leucine, and isoleucine (ath00280, *p*<0.0001), glycolysis (ath00010, *p*<0.05), photosynthesis (ath00195, *p*<0.05), lysine degradation (ath00310, *p*<0.05), and propanoate metabolism (ath00640, *p*<0.05; Supplementary Figure S5D).

There were 965 DEGs only expressed in Group II. The DEGs were significantly enriched into the GO terms anion transport (GO: 0006820, *p*<0.0001), inorganic anion transport (GO: 0015698, *p*<0.001), organic anion transport (GO: 0015711, *p*<0.01), organic acid transport (GO: 0015849, *p*<0.01), and carboxylic acid transport (GO: 0046942, *p*<0.01) in the BP categories; and anion transmembrane transport activity (GO: 0008509, *p*<0.0001), phosphoric ester hydrolase activity (GO: 0042587, *p*<0.0001), peroxidase activity (GO: 0004601, *p*<0.01), oxidoreductase activity, acting on peroxide as acceptor (GO: 0016684, *p*<0.01), antioxidant activity (GO: 0016209, *p*<0.01), and active transmembrane transporter activity (GO: 0022804, *p*<0.01) in the MF categories (Supplementary Figure S6A). Enriched KEGG showed that the DEGs enrichment analysis were mapped onto alanine, aspartate and glutamate metabolism (ath00250, *p*<0.05), phosphatidylinositol signaling system (ath04070, *p*<0.05), phenylpropanoid biosynthesis (ath00940, *p*<0.05), inositol phosphate metabolism (ath00562, *p*<0.05), nitrogen metabolism (ath00910, *p*<0.05), and carotenoid biosynthesis (ath00906, *p*<0.05; Supplementary Figure S6B).

### Co-expression Network Analysis by WGCNA

**Figure 6:**
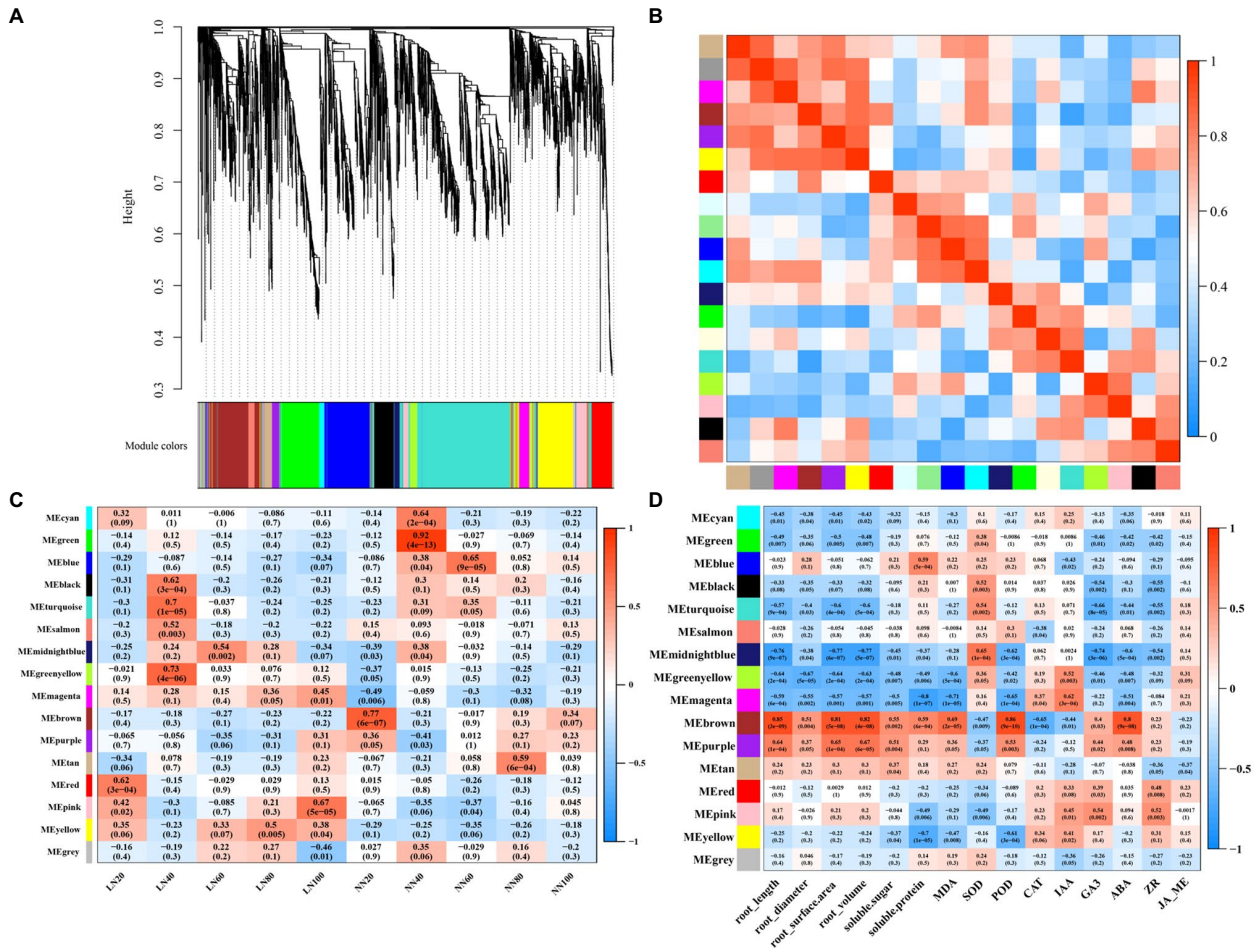
Weighted gene co-expression network analysis (WGCNA) for all DEGs. **(A)** Clustering diagram of all expressed genes together with assigned module colors, **(B)** the correlation between modules, **(C)** the relationship between modules and samples, and **(D)** the module-trait association.

To further represent a global view of transcriptional patterns throughout the growth period, we applied a WGCNA to define clusters of coregulated genes throughout the growing season. We obtained a total of 16 transcript expression (Figure 6). The module “MEmagenta” was positively correlated with low nitrogen treatment, but it was negatively correlated with normal nitrogen treatment. “MEmagenta” had significantly enriched GO functions, such as protein folding (GO: 0006457, *p*<0.0001), dicarboxylic acid transport (GO: 0006835, *p*<0.001), C_4_-dicarboxylate transport (GO: 0006457, *p*<0.001), ribosome binding (GO: 0043022, *p*<0.001), uridylyltransferase activity (GO: 0070569, *p*<0.001), and ribonucleoprotein complex binding (GO: 0043021, *p*<0.001), and KEGG analysis showed that the DEGs were mapped onto beta-alanine metabolism (ath00410, *p*<0.05; Supplementary Figure S7). Correlations were computed between each module and phenotypic trait. Interestingly, significantly correlations were found between each trait and at least one module. The module “MEbrown” had significantly positive correlation with root traits, soluble sugar, and protein, MDA, and ABA content, while “MEmagenta” and “MEgreenyellow” had significantly negative correlation with these traits. The DEGs of “MEbrown” were significantly enriched in the GO terms metal ion transport (GO: 0030001, *p*<0.01), anion transport (GO: 0006820, *p*<0.01), response to oxidative stress (GO: 0006979, *p*<0.05), inorganic diphosphatase activity (GO: 0004427, *p*<0.001), and acid phosphatase activity (GO: 0003993, *p*<0.05), and KEGG analysis showed that the DEGs were mapped onto circadian rhythm (ath04712, *p*<0.01) and plant hormone signal transduction (ath04075, *p*<0.05). Subsequently, we identified 5 hub genes in the module “MEmagenta”: *Ghir*_*A05G025560*, *Ghir*_*A11G025240*, *Ghir*_*A03G004210*, *Ghir*_*A02G001380*, and *Ghir*_*D02G002510* (Supplementary Figure S8).

### Phytohormone-Related Gene Analysis

**Figure 7:**
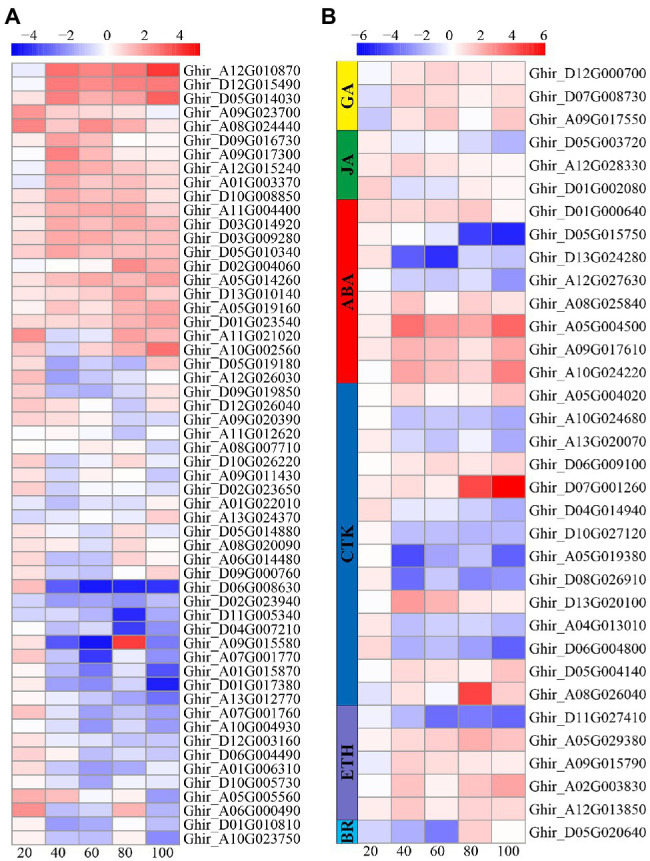
Heatmap illustrating differential expression of auxin **(A)**, gibberellic acid (GA), jasmonic acid (JA), ABA, cytokinein (CTK), ethylene (ETH), and brassinolide (BR; **B**) at 20, 40, 60, 80, and 100days after emergence. Different scale color represents the size of log_2_ fold change value.

A total of 90 DEGs involved in plant hormone pathways were identified, including 56 IAA pathway genes, 14 cytokinein (CTK) pathway genes, eight ABA pathway genes, three gibberellic acid (GA) genes, three JA pathway genes, five ETH pathway genes, and one brassinolide (BR) pathway gene (Figure 7). Interestingly, more than half of the auxin and CTK pathway genes were upregulated at 20 DAE, while most of the auxin and CTK pathway genes were downregulated at the other four phases. On the contrary, three GA pathway genes were down regulated at 20 DAE, but upregulated after 20 DAE. Four ETH genes were upregulated and one was downregulated after 20 DAE. There were three ABA genes were downregulated and five were upregulated after 20 DAE. One BR gene was downregulated at 20, 40, and 60 DAE, but upregulated at 80 and 100 DAE. Three JA genes were upregulated at 20 DAE.

### ROS Detoxification System Gene Analysis

**Figure 8:**
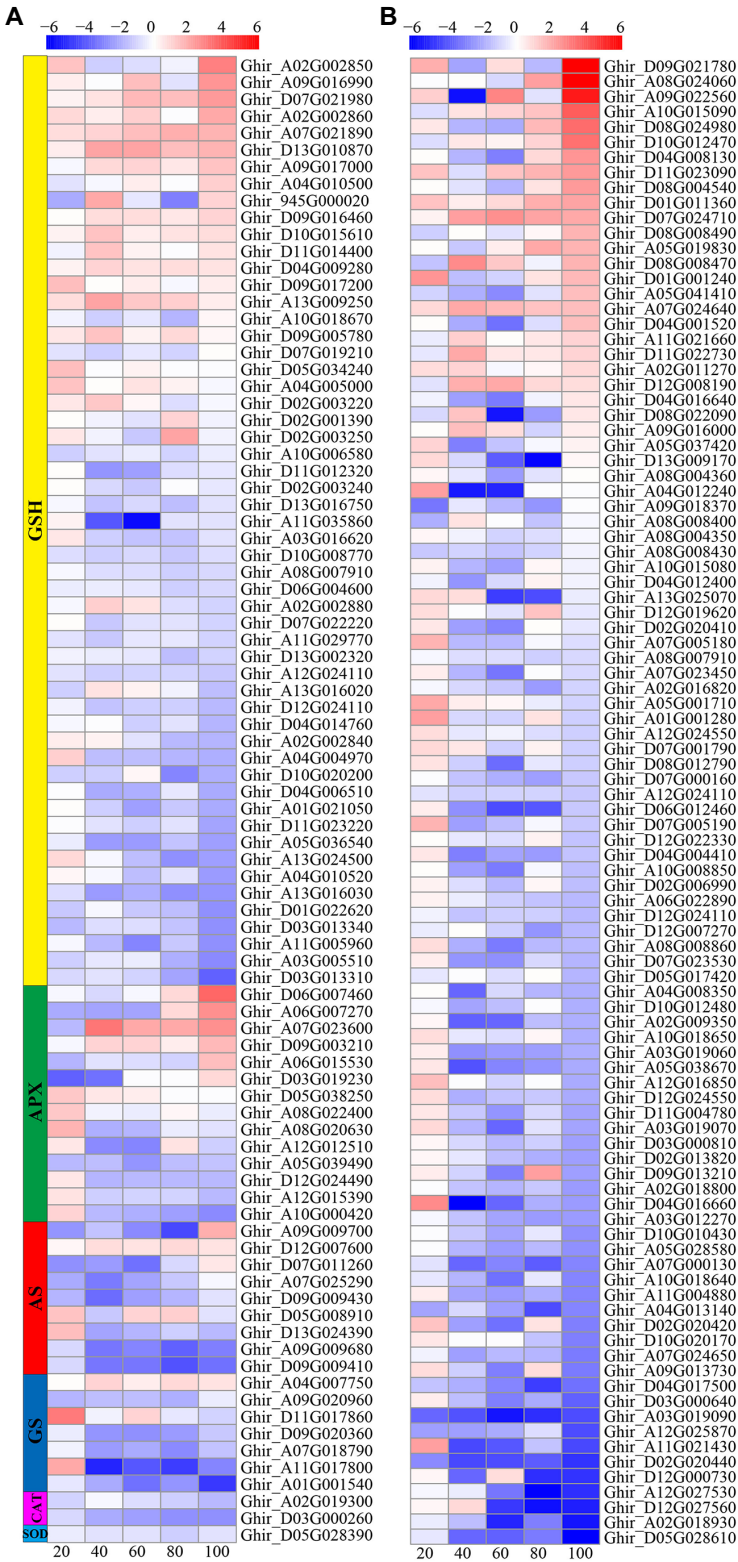
Heat-map illustrating differential expression of glutathione (GSH), ascorbate peroxidase (APX), asparagine synthetase (AS), glutathione synthetase (GS), CAT, and SOD **(A)**, and POD **(B)** at 20, 40, 60, 80, and 100days after emergence. Different scale color represents the size of log_2_ fold change value.

The reactive oxygen species scavenging system consists of a variety of enzymes and non-enzymatic antioxidants. In this study, a total of 187 DEGs involved in ROS scavenging pathways were identified, including 99 POD related genes, 55 glutathione (GSH) related genes, 14 ascorbate peroxidase (APX) related genes, nine asparagine synthetase (AS) related genes, seven glutathione synthetase (GS) related genes, two CAT related genes, and one SOD related gene (Figure 8). Most of the POD, AS, and APX related genes were downregulated; whereas, a small number of genes were upregulated at 80 and 100 DAE. About one-third of the GSH related genes were upregulated and two-third of the GSH related genes downregulated. Most GS, CAT, and SOD related genes were downregulated.

### Cell Cycle Pathway Analysis

**Figure 9:**
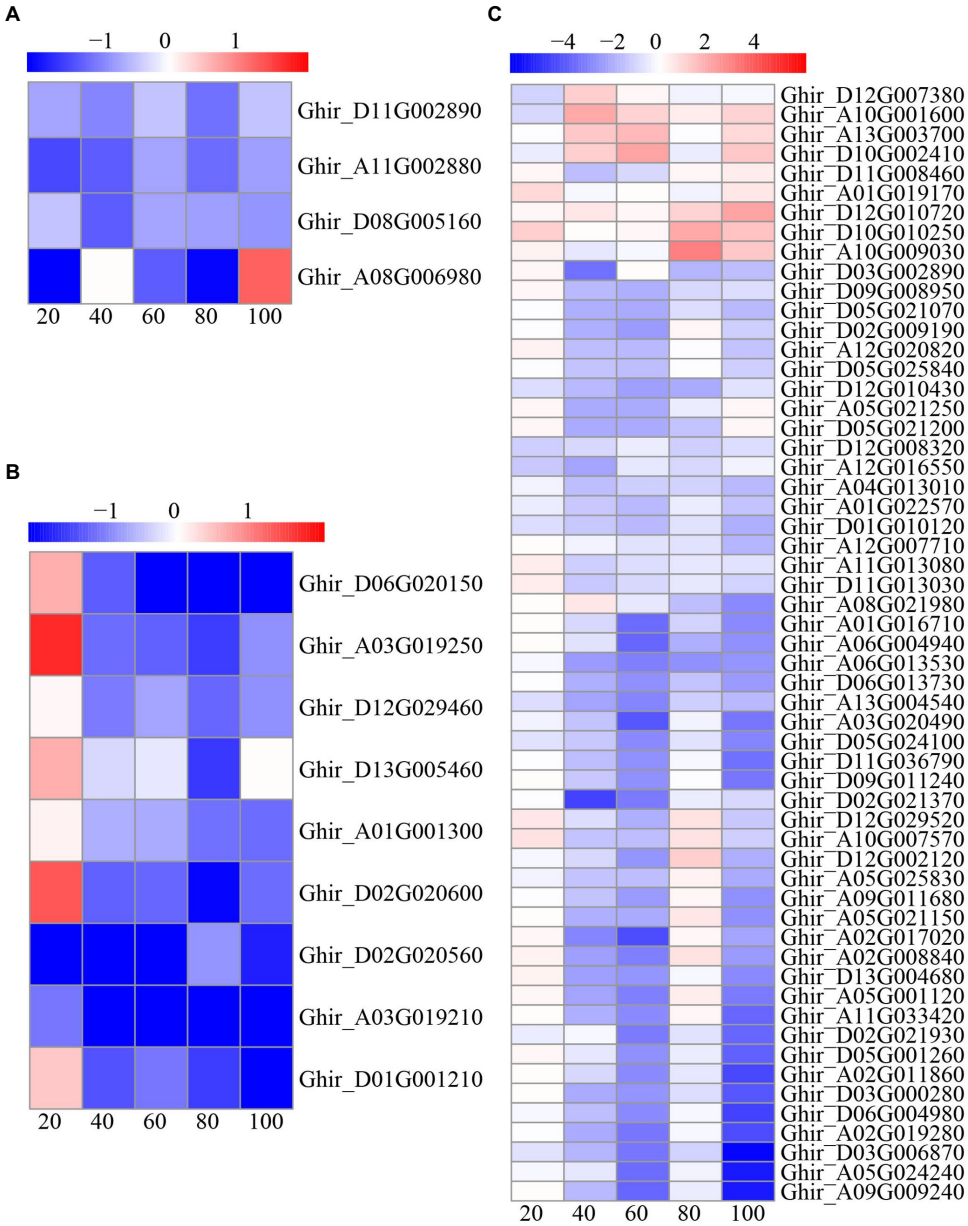
Heat-map illustrating differential expression of apoptosis **(A)**, caspase **(B)**, and cyclin (CYC; **C**) at 20, 40, 60, 80, and 100days after emergence. Different scale color represents the size of log_2_ fold change value.

In this study, a total of 70 DEGs were found to be involved in the cell cycle, including 57 cyclin (CYC) genes, nine caspase genes, and four apoptosis genes (Figure 9). Interestingly, most of the CYC and caspase genes were upregulated at 20 DAE, while most of the CYC and caspase genes were downregulated at the other four phases. All of the apoptosis genes were downregulated, except for *Ghir*_*A08G006980* at 40 and 100 DAE.

### Carbon and Nitrogen Metabolism Gene Analysis

**Figure 10:**
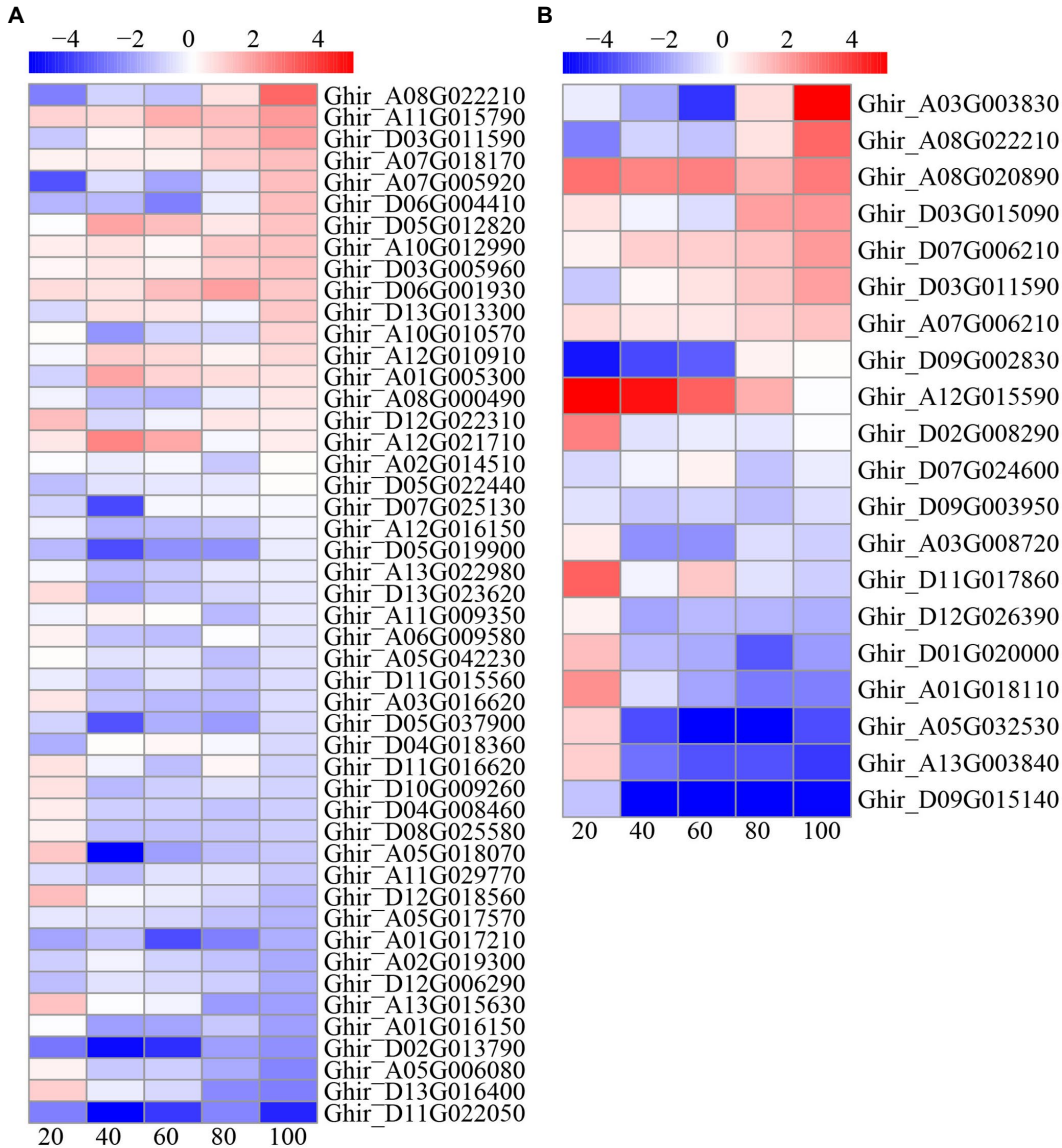
Heatmap illustrating differential expression of carbon **(A)** and nitrogen **(B)** metabolism at 20, 40, 60, 80, and 100days after emergence. Different scale color represents the size of log_2_ fold change value.

There were 48 and 21 DEGs involved in carbon and nitrogen metabolism, respectively (Figure 10). For carbon metabolism, nearly half of DEGs were upregulated at 20 and 100 DAE, while most genes were downregulated at 40, 60, and 80 DAE. On the contrary, most of the nitrogen metabolism genes were upregulated at 20 DAE, but most genes were downregulated at 40 and 60 DAE. Interestingly, nearly half of nitrogen metabolism genes were upregulated at 80 and 100 DAE.

### Transcription Factor Genes’ Analysis

**Figure 11:**
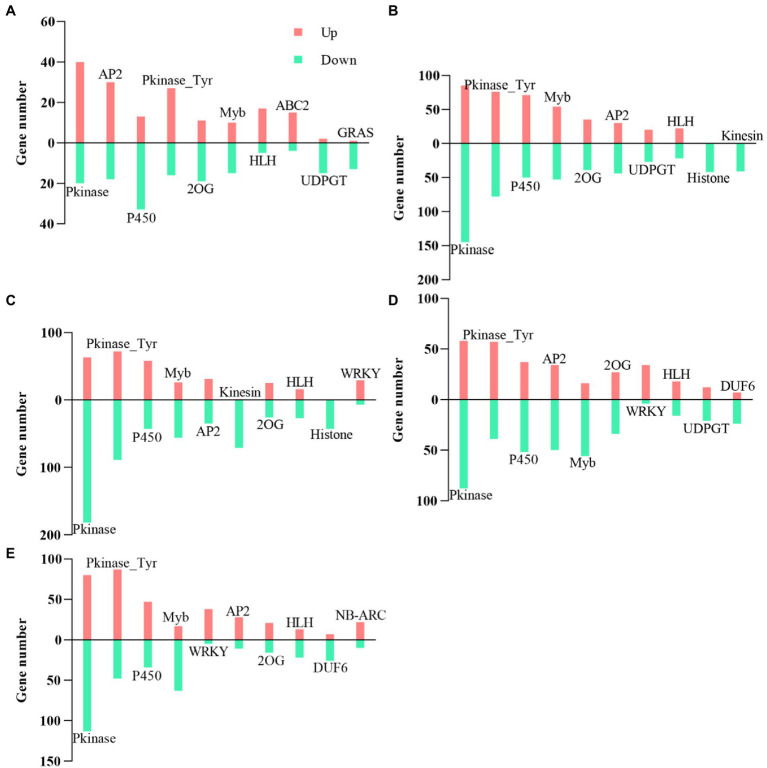
Top 10 transcription factor (TF) statistics. Top 10 TFs at 20DAE **(A)**, 40 DAE **(B)**, 60 DAE **(C)**, 80 DAE **(D)**, and 100 DAE **(E)**. 2OG, 2OG-FeII_Oxy; Myb, Myb_DNA-binding; ABC2, ABC2_membrane.

A total of 6,000 DEGs encoding the TF family were identified in cotton roots. Here, we focused on the top 10 TFs families during each period. We found that the TFs families’ Pkinase, P450, Pkinase_Tyr, 2OG-FeII_Oxy (2OG), and Myb_DNA-binding (Myb) were identified in five periods (Figure 11). There were more downregulated genes among these five TF family genes than upregulated genes, except for Pkinase genes at 20 DAE, P450 and Myb genes at 40 DAE, and Pkinase_Tyr genes at 20, 80, and 100 DAE. Meanwhile, the number of upregulated genes of HLH and ABC2 at 20 DAE, WRKY at 60, 80, and 100 DAE, and NB-ARC and AP2 at 100 DAE was much greater than the number of downregulated genes. In contrast, the number of downregulated genes of UDPGT and GRAS at 20 DAE, Histone and Kinesin at 40 and 60 DAE, Myb at 60, 80, and 100 DAE, and DUF6 at 80 and 100 DAE was much greater than the number of upregulated genes.

### Validation of DEGs by qRT-PCR

To validate the RNA-Seq data, qrRT-PCR analysis was performed to examine the expression profiles of 10 randomly chosen DEGs. As a result, the expression levels of these selected genes showed a strong correlation with the data of RNA-Seq data (*R*^2^=0.8691, *p*<0.0001; Supplementary Figure S9).

## Discussion

### Nitrogen Stress Delays Root Senescence

Nitrogen is the basis for plant growth and development. A lack of nitrogen leads to reduced photosynthetic properties, root growth inhibition, and organ senescence, ultimately affecting the yield and quality (Stefan and Urs, 2002; Sandrine, 2018). Many studies have demonstrated that nitrogen starvation can induce leaf senescence (Diaz et al., 2008; Dong et al., 2012; Yang et al., 2016; Kitonyo et al., 2018; Zakari et al., 2020). In the present study, leaf senescence was assessed by leaf color change and SPAD value (Chen and Dong, 2016). Both of these indicators showed that nitrogen deficiency induced premature senescence in leaves (Figure 1; Supplementary Figure S1). These results were generally consistent with previous studies.

Compared with leaves, little research has been conducted on root senescence in response to nitrogen stress. A large body of evidence has demonstrated that nitrogen starvation can increase the lifespan of roots (Burton et al., 2000; Eissenstat et al., 2000). However, conflicting reports were also obtained by other researchers (Aber et al., 1985). In general, the degree of root senescence can be determined by the change in root color, roots were regarded as dead when the roots became entirely black (Hendrick and Pregitzer, 1992). Our results showed that the root color under nitrogen treatment was lighter than that under normal nitrogen treatment at 80 and 100 DAE (Figure 1). Therefore, we deduced that nitrogen starvation delayed root senescence. Low nitrogen treatment significantly reduced the total root length (Supplementary Figure S2). We speculated that plants chose to prolong root lifespan instead of producing new roots under nitrogen depletion. This was mainly due to the fact that building new roots would consume a large amount of metabolic energy (Schoettle et al., 1994; Eissenstat et al., 2000; Janssens et al., 2002), which would result in the inhibition of aboveground growth.

Taken together, low nitrogen (extreme nitrogen deficiency) induced leaf senescence while delaying root senescence. Fanello et al. (2019) demonstrated that roots of soybean are under the control of the aboveground parts until the final stage, and survive independently for some time following leaf senescence. Similarly, *Arabidopsis thaliana* roots maintain their respiratory activity until the final stage even when the aboveground tissues are fully withered (Fanello et al., 2017). In other words, the continued activity of root systems following leaf senescence suggests that roots may not undergo a clear-cut senescence process as leaves do.

### Low Nitrogen Delayed Root Senescence by Regulating the ROS Detoxification System

In plants, reactive oxygen species are by-products of aerobic metabolism including singlet oxygen (^1^O_2_), hydroxyl radicals (OH), superoxide anions ($O_{2}^{\cdot -})$, and hydrogen peroxide (H_2_O_2_; Apel and Hirt, 2004; Mittler et al., 2004; Dietz et al., 2016). The accumulation of ROS will aggravate membrane lipid peroxidation. MDA is one of the products of membrane lipid peroxidation, further lead to irreversible DNA damage, and premature cell death (Xie et al., 2014). Some evidence demonstrates that ROS participate in senescence initiation and signal transduction (Ye et al., 2000; Saeid et al., 2003; Zimmermann et al., 2010). Plants employ an ROS scavenging system to protect them from oxidative damage (Fischer, 2012) that includes enzymes such as CAT, SOD, POD, and APX, and antioxidant molecules including GSH, ascorbate, and others (Apel and Hirt, 2004).

In this study, the POD activity was significantly inhibited by low nitrogen treatment (Figure 2). Meanwhile, we identified 99 POD-related DEGs, most of which were downregulated (Figure 8). In contrast, the activity of SOD at 100 DAE and CAT activity at 60, 80, and 100 DAE under low nitrogen treatment were higher than that of normal nitrogen treatment (Figure 2). This may be the reason why MDA content was reduced by low nitrogen treatment at 60, 80, and 100 DAE. However, only one SOD- and two CAT-related genes were identified, all of which were downregulated. The transcriptome data was not well correlated with the results of physiological analysis. This might have been because few DEGs were identified, or due to post-transcriptional and post-translational modification (Chen et al., 2020b). In addition, we identified 55 GSH-related DEGs (Figure 8). Although more than half of these genes were downregulated, a small percentage was markedly upregulated such as *HSP26-A* (Ghir_D13G010870) and *GSTU8* (Ghir_A09G017000). We found that APX genes *MIOX2* (Ghir_A06G007270) and *MIOX4* (Ghir_D06G007460) were significantly upregulated at 80 and 100 DAE. Previously, upregulation of *MIOX* family genes has been reported at rice under drought stress, which significantly improved the activity of ROS scavenging enzymes (Duan et al., 2012). Therefore, we speculated that cotton roots enhanced the activity of SOD and CAT at the late growth stage, and that SOD and CAT combined with APX and GSH to scavenge ROS and reduce the MDA content, eventually delaying the senescence of roots under nitrogen starvation.

### Low Nitrogen Delayed Root Senescence by Modulating Phytohormones

The relationship between hormones and plant senescence has long been studied (Richmond and Lang, 1957; Stoddart and Thomas, 1982). Many studies have shown that the genes coding ABA synthesis and signaling are upregulated during leaf senescence (Buchanan et al., 2005; Jukanti et al., 2008). In this study, we found that the ABA content under low nitrogen treatment was significantly higher than that under normal nitrogen treatment at 20 DAE, while the opposite results were shown at 80 and 100 DAE (Figure 3). Meanwhile, the relative quantitative expression of ABA synthesis genes *NCED1* and *NCED6* and ABA catabolic genes *CYP707A2* and *CYP707A4* under low nitrogen treatment were downregulated, except for at 80 DAE (Figure 4). We identified eight ABA related DEGs, with three downregulated and five upregulated (Figure 7). ABA receptor gene *PYL12* (Ghir_D05G015750) was significantly downregulated under low nitrogen treatment at 80 and 100 DAE (Figure 7). Zhao et al. (2016) demonstrated that *PYL9* promoted ABA-induced leaf senescence and the *PYL9* single mutant showed delayed leaf senescence. Similarly, ETH (Weaver et al., 1998), BR (Graaff et al., 2006), and jasmonate (JA; Rossato et al., 2002) promoted senescence. Our transcriptome results showed that BR-related DEG *BKI1* (Ghir_D05G020640) was downregulated at 20, 40, and 60 DAE, while it was upregulated at 80 and 100 DAE. *BKI1* was the negative regulator of BR signaling. In contrast, CTK can delay senescence because endogenous cytokinin levels decrease during leaf senescence and elevated cytokinin levels accompany delayed senescence (Dong et al., 2008; Zhao et al., 2013). In this study, there was no significant difference in ZR content between nitrogen treatments, except for at 60 DAE (Figure 3). Most of the DEGs identified to be involved in CTK dehydrogenase were downregulated (Figure 7). Compared with the normal nitrogen treatment, there was no significant reduction in CTK content under low nitrogen treatment due to the downregulated of CTK dehydrogenase genes.

However, the role of auxin (IAA) and gibberellin (GA) in regulating senescence was much less clear. Noh and Amasino (1999) showed that the expression of developmental senescence gene *SAG12* was downregulated in *Arabidopsis* leaves induced by 5μM IAA. In Alstroemeria, GA4 levels dramatically reduced after the onset of senescence (Kappers et al., 2010), but the experiments did not determine whether the drop in GA content was the cause or the consequence of senescence onset. In the current study, low nitrogen treatment significantly reduced the IAA content, while there was no significant impact on GA3, except for at 40 DAE (Figure 3). Consistently, most IAA-related DEGs identified here were downregulated, whereas the GA related DEGs were upregulated, except for at 20 DAE. Hormones play crucial roles in senescence regulation, but there is extensive crosstalk between different hormones (Fischer, 2012). In this study, the low nitrogen treatment reduced root diameter and length by means of decreasing the IAA content, which reduced carbon and energy costs (Lynch, 2007). This may lead to a delay in root senescence under low nitrogen. Therefore, we concluded that auxin plays a negative regulatory role in root senescence.

### Low Nitrogen Delayed Root Senescence by Modulating Apoptosis and Cell Cycle Pathway

Apoptosis, a type of programmed cell death, plays an integral role in plant. It is controlled by cellular oxidative status, hormones, and NDA methylation and plays vital roles in plant development and adaptive responses (Vanyushin et al., 2004; Chen et al., 2021). Research has shown that leaf senescence and apoptosis are linked by the downstream of the ethylene trifurcate pathway (Jin et al., 2009). In recent years, increasing evidence has suggested the existence of caspase-like activity in plant apoptosis. Interestingly, we found that four apoptosis-related DEGs were downregulated, and caspase-related DEGs were also downregulated except 20 DAE (Figure 9). Thus, we speculated that apoptosis-related genes played crucial roles in cotton root senescence. In other words, this result confirmed that low nitrogen treatment delayed cotton root senescence.

Cell cycle regulators can affect the growth, division, and differentiation of plant cells by influencing the cell cycle, further regulating plant growth and development (He and Zheng, 2010). CYC is one of the main cell cycle regulators (Andersen et al., 2008). In this study, most of the CYC-related genes were downregulated, except for at 20 DAE (Figure 9). This result may verify that plants choose to prolong the lifespan of their roots instead of producing new roots under nitrogen deficiency.

### Low Nitrogen Delayed Root Senescence by Regulating the Expression of TFs

To date, *NAC* and *WRKY* TFs have been found to be involved in the regulation of leaf senescence (Bakshi and Oelmüller, 2014; Chen et al., 2017). Kong et al. (2013a) identified 11 *NAC* and eight *WRKY* TF genes were upregulated in early senescence in cotton leaves. *WRKY* TFs regulated leaf senescence by interacting with proteins *via* GA, JA, IAA, and salicylic acid (Jiang et al., 2014). Moreover, numerous genes, including *C2H2*, *AP2*, *MYB*, and *GRAS* TFs, were upregulated during senescence (Chen et al., 2002; Buchanan et al., 2005; Guo et al., 2010; Breeze et al., 2011). Pourtau et al. (2006) demonstrated that the gene *PAP1* of *MYB* was upregulated under low nitrogen conditions in *Arabidopsis* and induced senescence. However, we are far from a comprehensive understanding of all TFs involved in plant senescence.

In the current study, we found that most of the genes among the *WRKY* TFs were upregulated at 60, 80, and 100 DAE, and the genes were positive regulators of plant stress tolerance (Figure 11). *WRKY22* was significantly upregulated under low nitrogen treatment (Ghir_A12G024530), which could delay senescence (Miao et al., 2007). Whereas *WRKY53* was downregulated (Ghir_A12G024820), which could promote senescence (Miao et al., 2004). Moreover, we identified five *WRKY75* and three *WRKY72* TF genes were significantly upregulated under low nitrogen treatment. Hence, we speculated that these two *WRKY* TFs may play critical roles in delay root senescence. In addition, we found that most of *MYB* TF genes of low nitrogen treatment were downregulated at 60, 80, and 100 DAE.

### The Underlying Pathway of Low Nitrogen Regulates Root Senescence

Weighted gene co-expression network analysis revealed that the module “MEmagenta” had significant negative correlation with MDA and ABA contents, while it had positive correlation with low nitrogen treatment (Figure 6). Therefore, we speculated that “MEmagenta” may play a crucial role in delaying root senescence under low nitrogen treatment. Further GO enrichment analysis showed that “MEmagenta” genes were significantly enriched in protein folding (GO: 0006457, *p*<0.0001), anion transport (GO: 0006820, *p*<0.001), dicarboxylic acid transport (GO: 0006835, *p*<0.001), ribosome binding (GO: 0043022, *p*<0.001), and uridylyltransferase activity (GO: 0070569, *p*<0.001), and KEGG mapped these genes onto beta-alanine metabolism (ath00410, *p*<0.05). Meanwhile, we identified five hub genes in the module “MEmagenta.” Hence, all of these pathways and hub genes merit further study and may play crucial roles in the regulation of cotton root senescence.

## Conclusion

**Figure 12:**
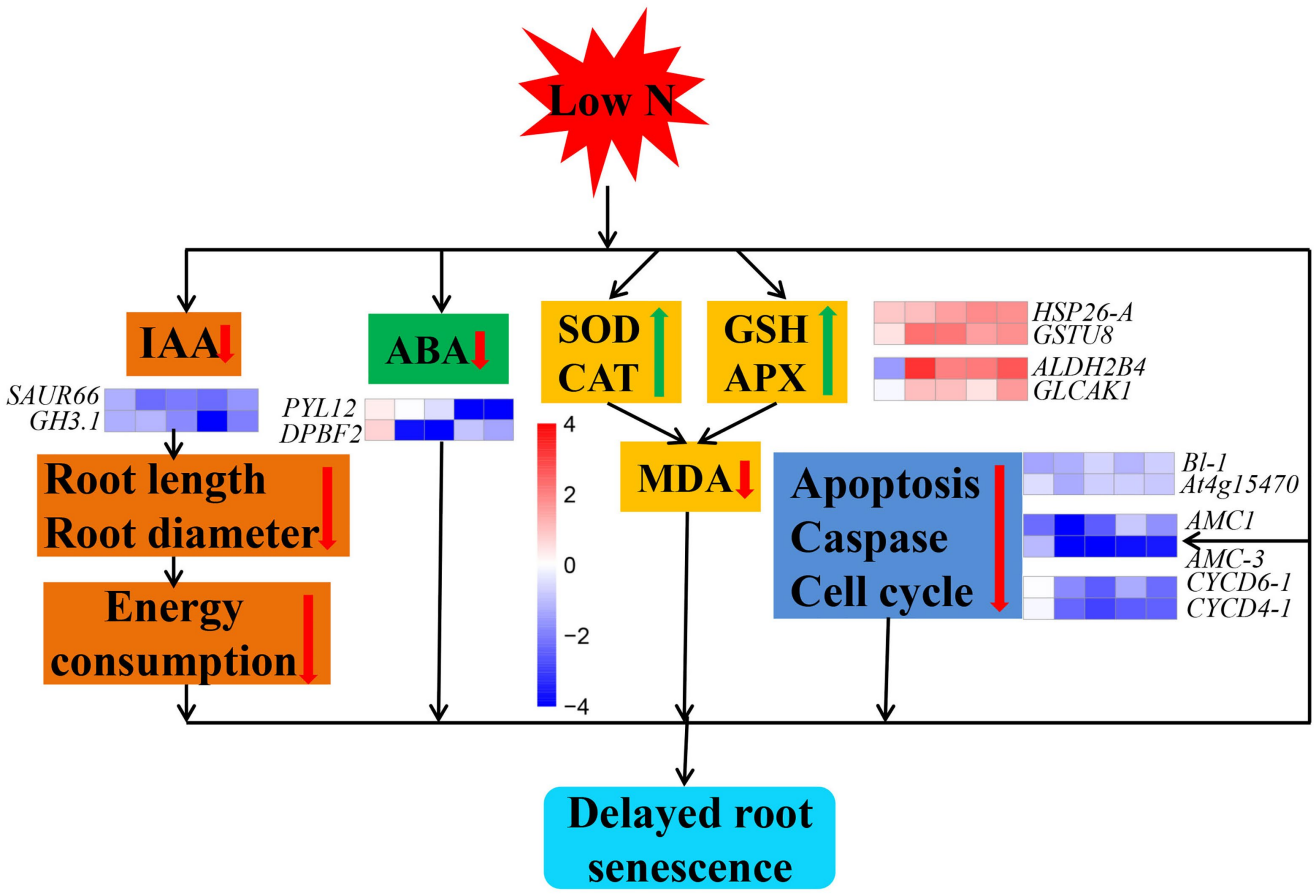
Model showing the mechanism of cotton root senescence under nitrogen stress. The red and green arrows represent a decrease and increase in an indicator, respectively. Cubes represent the value of log_2_ fold change (from left to right: comparisons between low nitrogen and normal nitrogen treatment at 20, 40, 60, 80, and 100 DAE). Red color indicates upregulated, while blue indicates downregulated.

Our investigation conducted in cotton indicated that low nitrogen delays root senescence through ways (Figure 12). First, cotton roots reduced IAA and ABA contents by downregulating expression of IAA and ABA pathway related key genes (Supplementary Table S3). Second, cotton roots promoting the activity of SOD, CAT, GSH, and APX by upregulating of antioxidase pathway related key genes. Finally, cotton roots downregulated expression of apoptosis, caspase, and cell cycle related key genes. In addition, we found that roots senescence might be negatively correlated with nitrogen content of root. Overall, we clarified the effect of low nitrogen on root senescence and the possible mechanism, which may serve as a basis for the study on root senescence in cotton and other crops.

## Data Availability Statement

The original contributions presented in the study are publicly available. This data can be found at: the Genome Sequence Archive of the National Genomics Data Center (NGDC) under accession number CRA004678 (https://bigd.big.ac.cn/gsa/browse/CRA004678).

## Author Contributions

LZ, LL, and CL conceived the idea, proposed the method, and acquired the financial support for the project leading to this publication. LZ, HS, YZ, and KZ contributed to the preparation of equipment and acquisition of the data. AL, ZB, and GW validated results. LZ wrote the paper. LL, JZ, and CL revised the paper. All authors contributed to the article and approved the submitted version.

## Funding

This study was supported by grants from the National Natural Science Foundation of China (No. 31871569), the National Key R&D Program of China (Nos. 2018YFD0100306 and 2017YFD0201900), Natural Science Foundation of Hebei Province (C2021204140), Graduate Innovation Funding Project of Hebei Province (CXZZBS2020089), and the Modern System of Agricultural Technology in Hebei Province (No. HBCT2018040201).

## Conflict of Interest

The authors declare that the research was conducted in the absence of any commercial or financial relationships that could be construed as a potential conflict of interest.

## Publisher’s Note

All claims expressed in this article are solely those of the authors and do not necessarily represent those of their affiliated organizations, or those of the publisher, the editors and the reviewers. Any product that may be evaluated in this article, or claim that may be made by its manufacturer, is not guaranteed or endorsed by the publisher.

## Footnotes

^1^ http://www.genome.jp/kegg/
