# Supplementary material for: Physiological and Comparative Transcriptomic Analysis Provide Insight Into Cotton (Gossypium hirsutum L.) Root Senescence in Response
Source: Front Plant Sci. 2021 Oct 18;12:748715. doi: 10.3389/fpls.2021.748715 (PMC8558499; doi:10.3389/fpls.2021.748715)
Supplement: Supplementary file 1 [file Data_Sheet_1.docx]

Supplementary Material

## Supplementary Figures


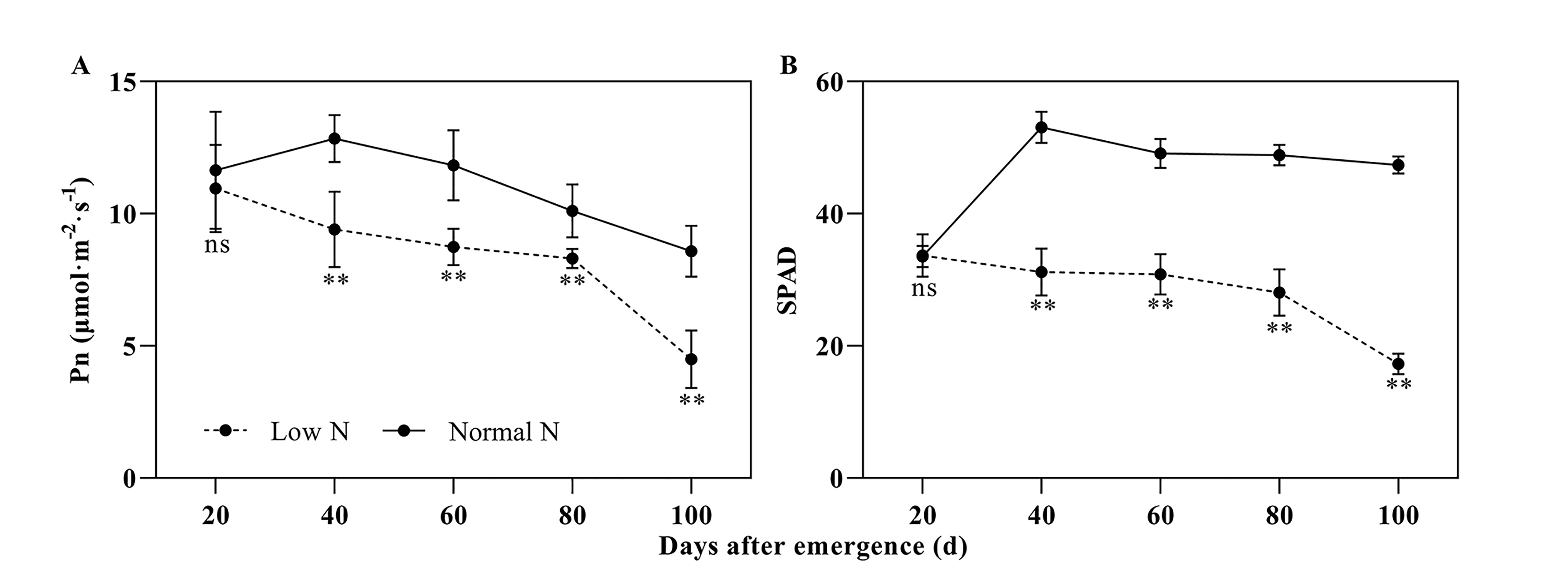


**FIGURE S1** (A) Net photosynthetic rate (Pn), and (B) soil and plant analyzer development (SPAD) value of cotton leaves under different nitrogen treatments. Depicted are the means of eight replicates± standard error. ns not significant (*p* > 0.05), ** *p*<0.01.*p*<0.01.


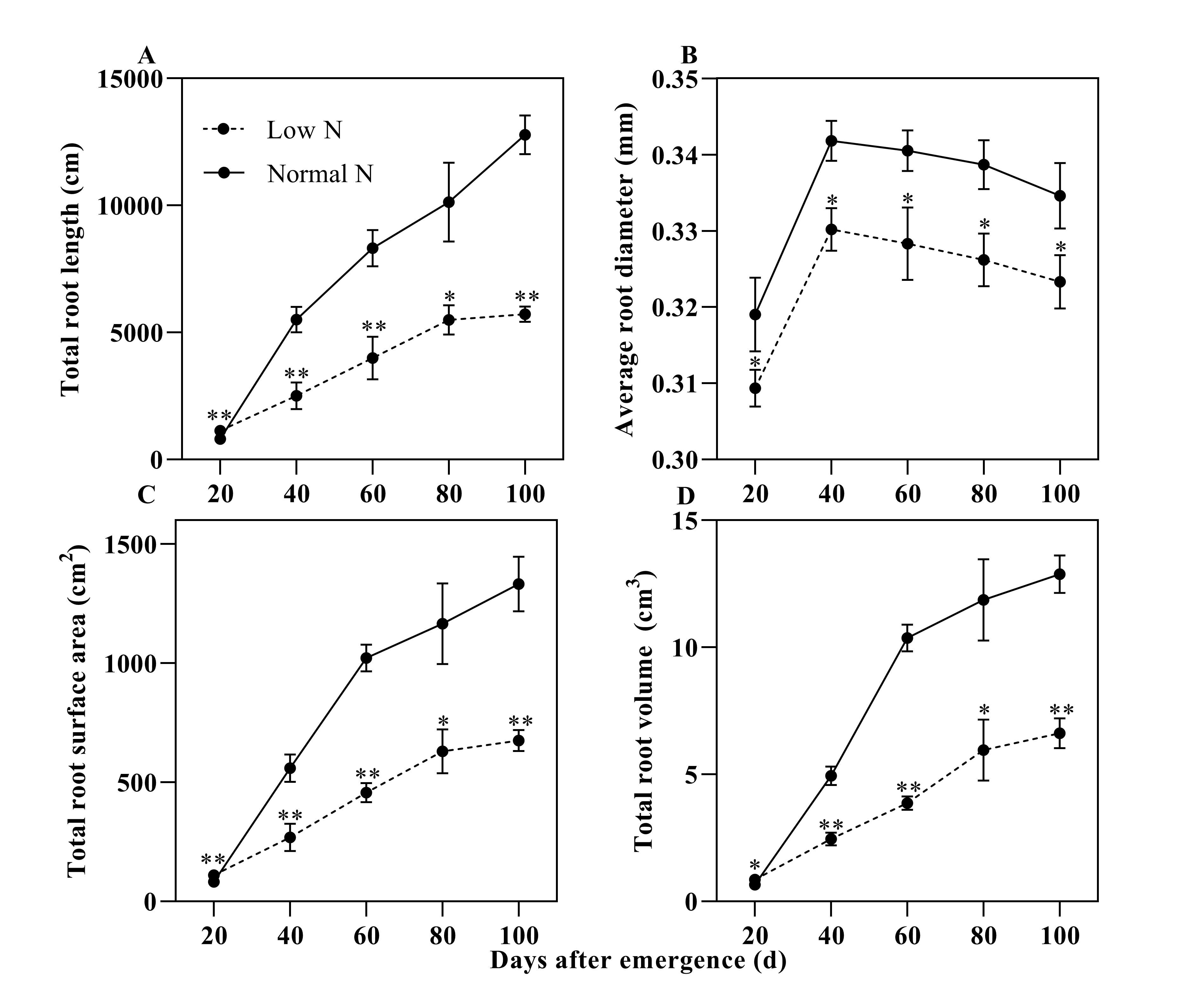


**FIGURE S2** Root parameters under different nitrogen treatments. **(A)** Total root length, **(B)** average root diameter, **(C)** total root surface area, **(D)** total root volume. Depicted are the means of three replicates ± standard error. * *p* < 0.05, ** *p* <0.01.


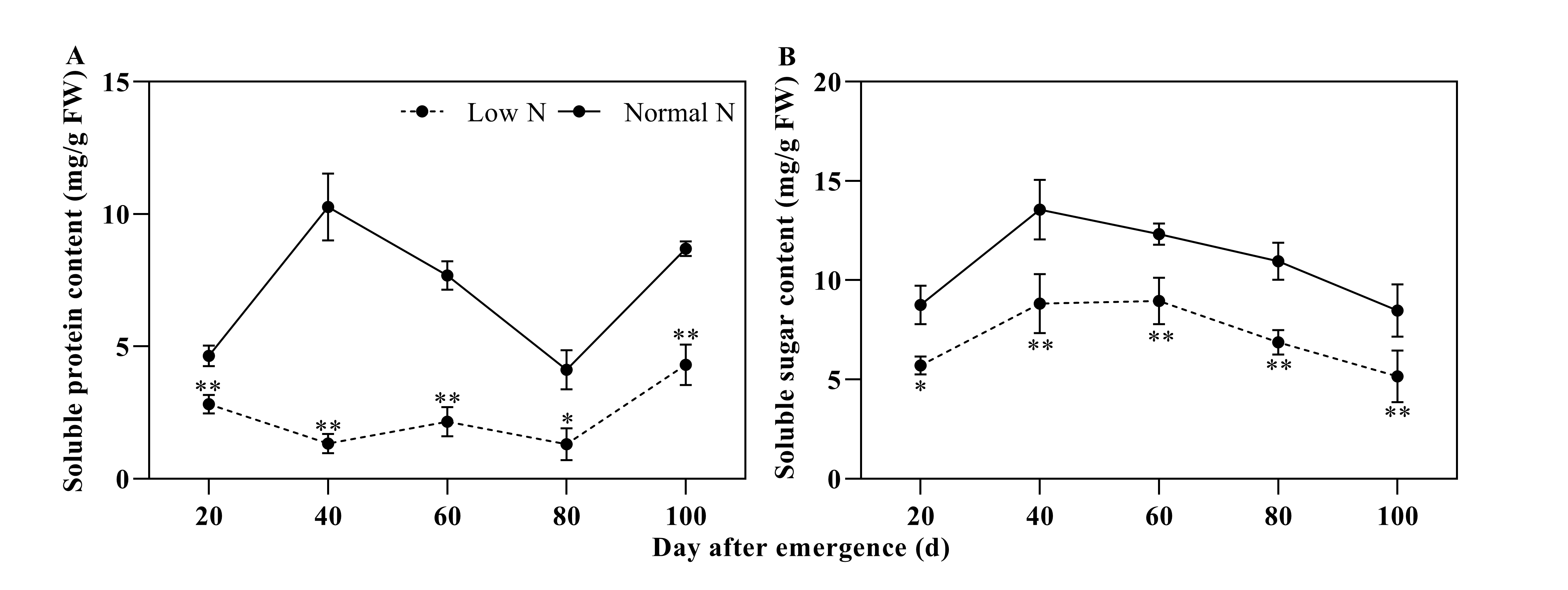


**FIGURE S3** **(A)** Soluble protein content, and **(B)** soluble sugar content of cotton roots under different nitrogen treatments. Depicted are the means of three replicates ± standard error. * *p* < 0.05, ** *p* < 0.01.


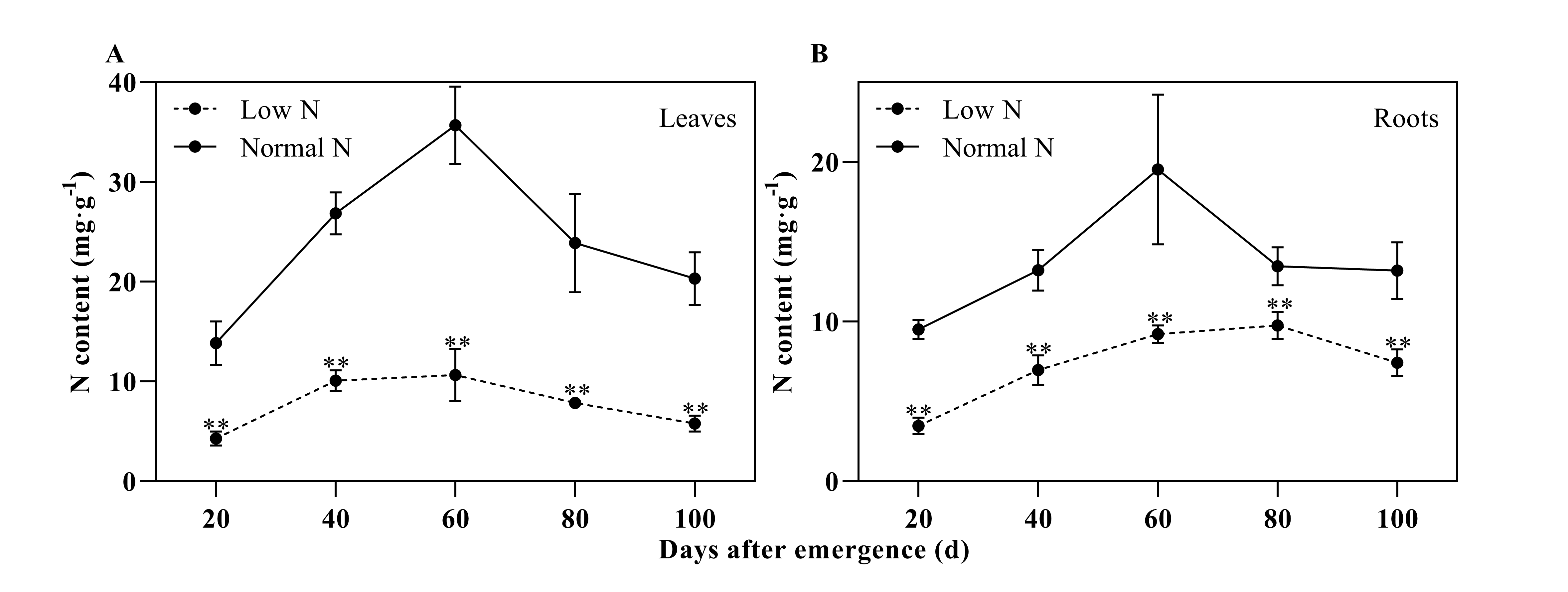


**FIGURE S4** Nitrogen content of leaves **(A)**, and roots **(B)** under different nitrogen treatments. Depicted are the means of three replicates ± standard error. ns, not significant (*p* > 0.05), ** *p* < 0.01.


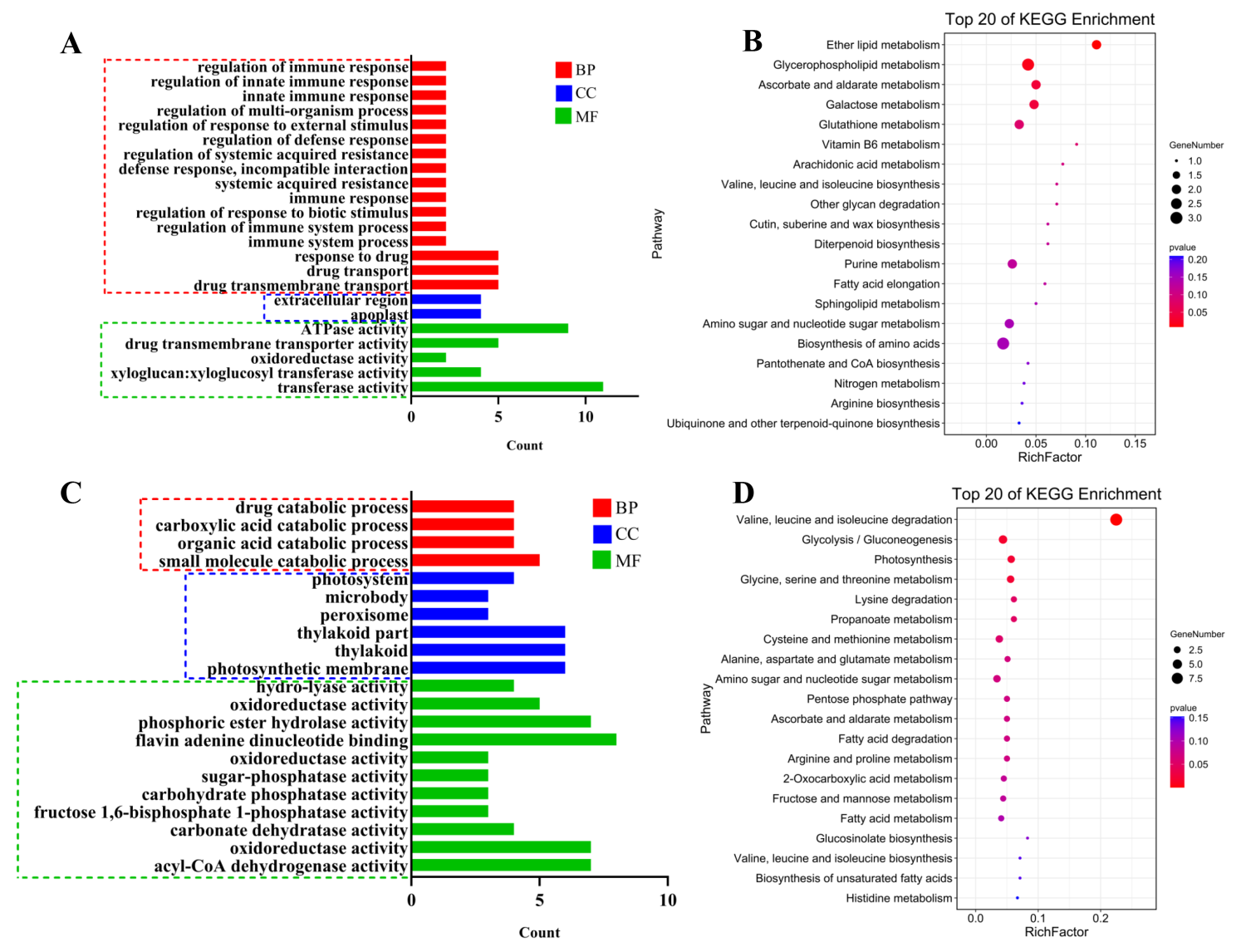


**FIGURE S5** GO enrichment analysis of the upregulated **(A)** and downregulated **(C)** DEGs only expressed in Group I. KEGG pathway enrichment analysis of the upregulated **(B)** and downregulated **(D)** DEGs only expressed in Group I. BP, biological process; CC, cellular component; MF, molecular function. GO, Gene ontology; KEGG, Kyoto Encyclopedia of Genes and Genomes; DEGs, differentially expressed genes.

**
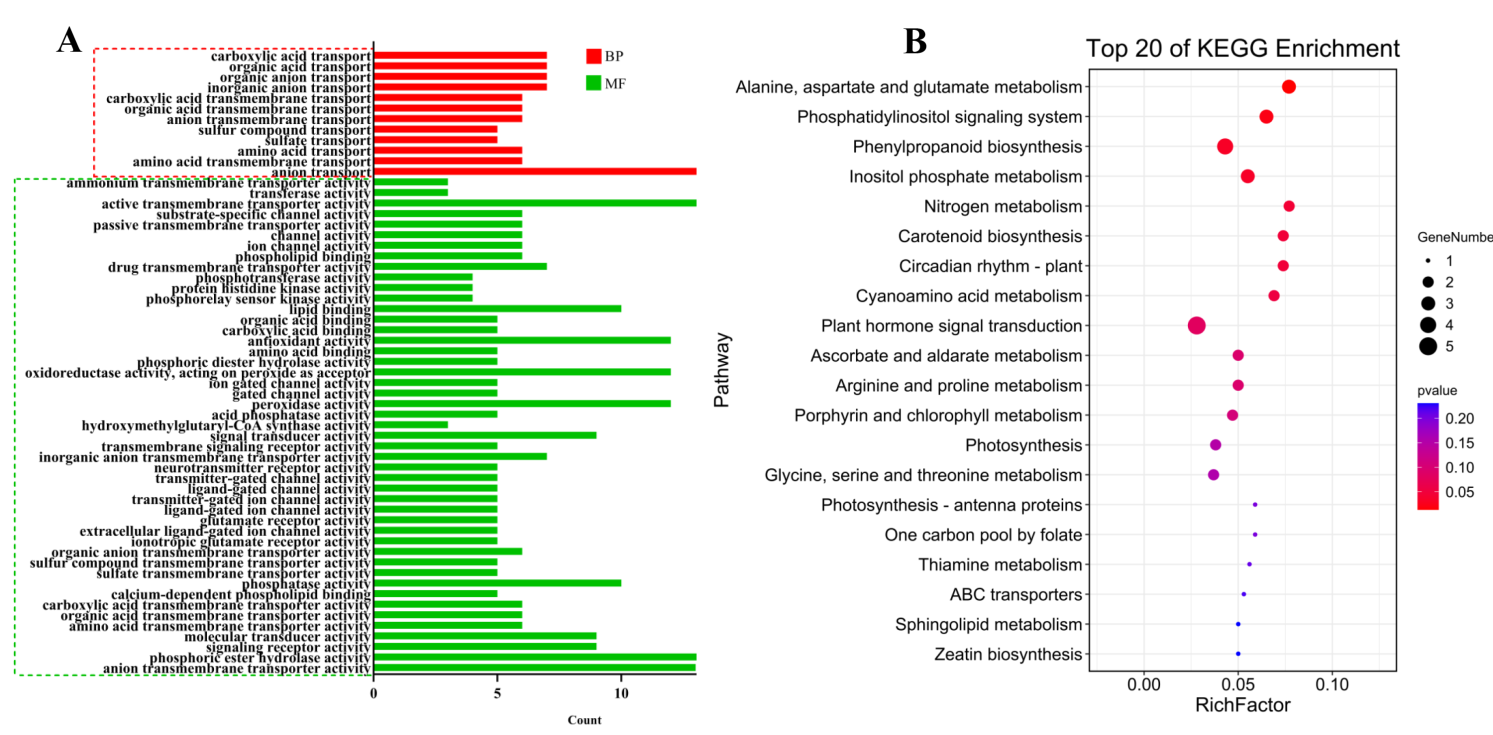
**

**FIGURE S6** **(A)** GO enrichment analysis of the DEGs only expressed in Group II. **(B)** KEGG pathway enrichment analysis of DEGs only expressed in Group II. BP, biological process; CC, cellular component; MF, molecular function. GO, Gene ontology; KEGG, Kyoto Encyclopedia of Genes and Genomes; DEGs, differentially expressed genes.

**
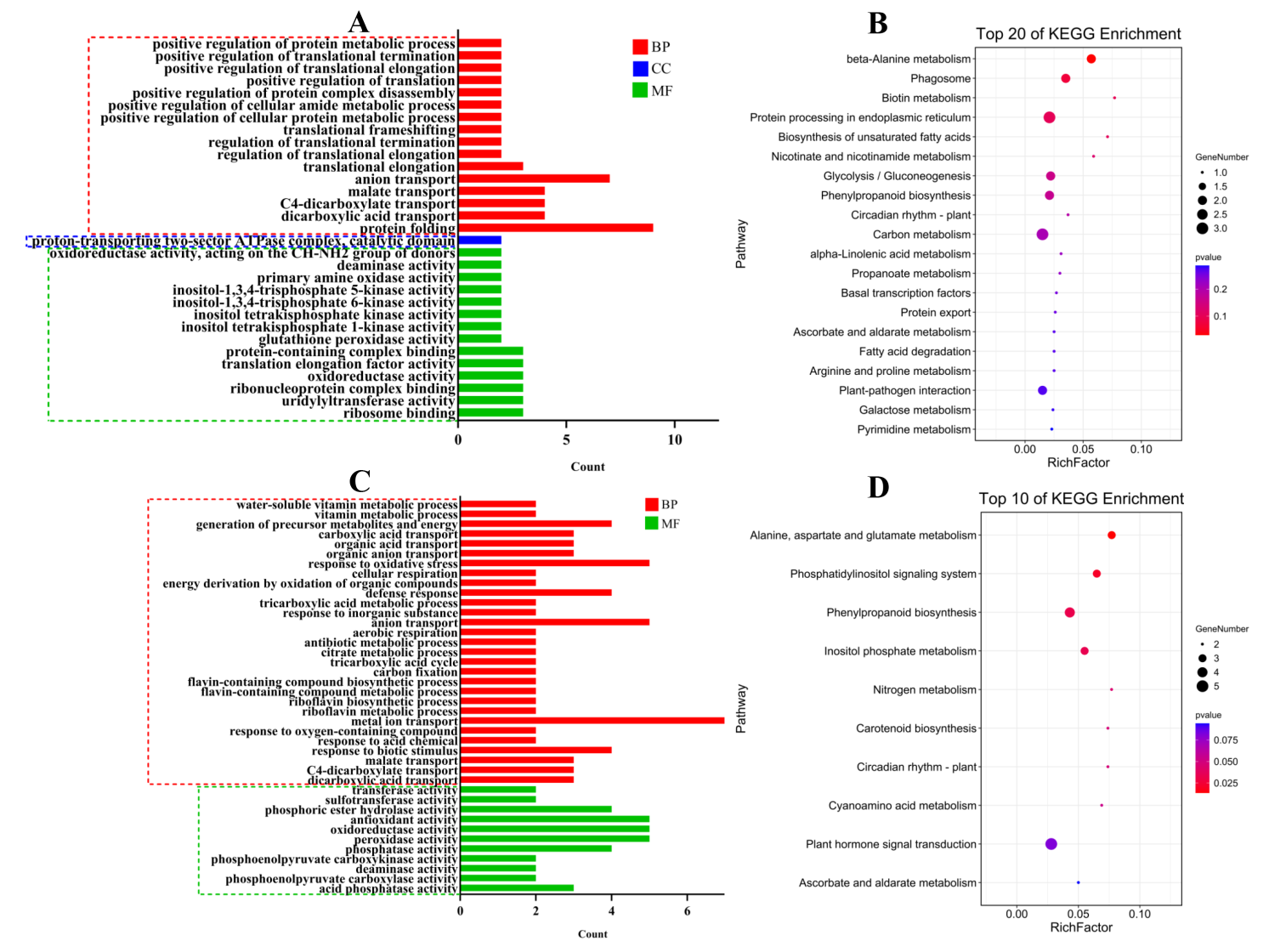
**

**FIGURE S7** GO enrichment analysis of the DEGs of “MEmagenta” **(A)** and “MEbrown” **(C)**. KEGG pathway enrichment analysis of the DEGs of “MEmagenta” **(B)** and “MEbrown” **(D)**. BP, biological process; CC, cellular component; MF, molecular function. GO, Gene ontology; KEGG, Kyoto Encyclopedia of Genes and Genomes; DEGs, differentially expressed genes.

**
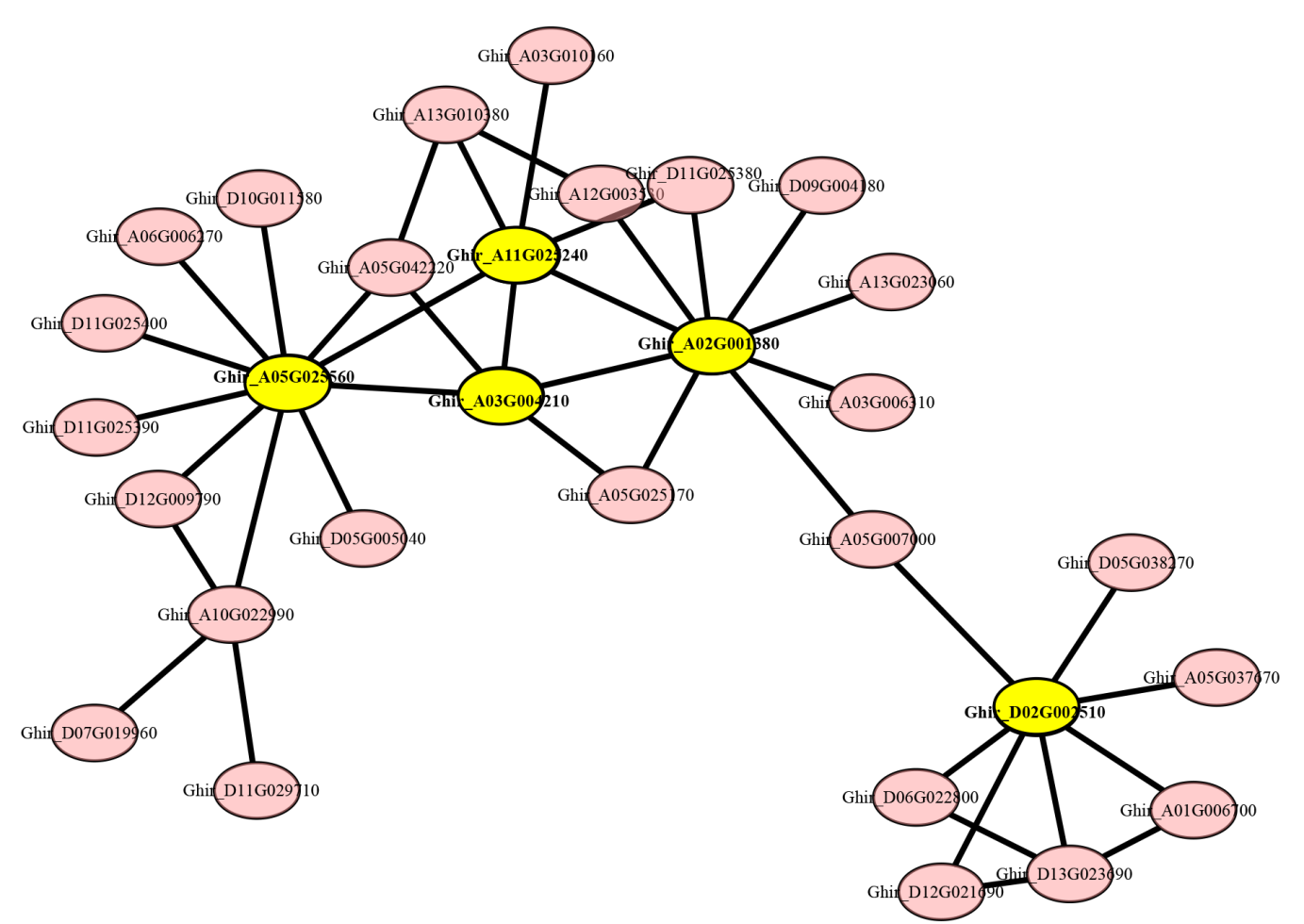
**

**FIGURE S8** Co-expression network of genes.

**
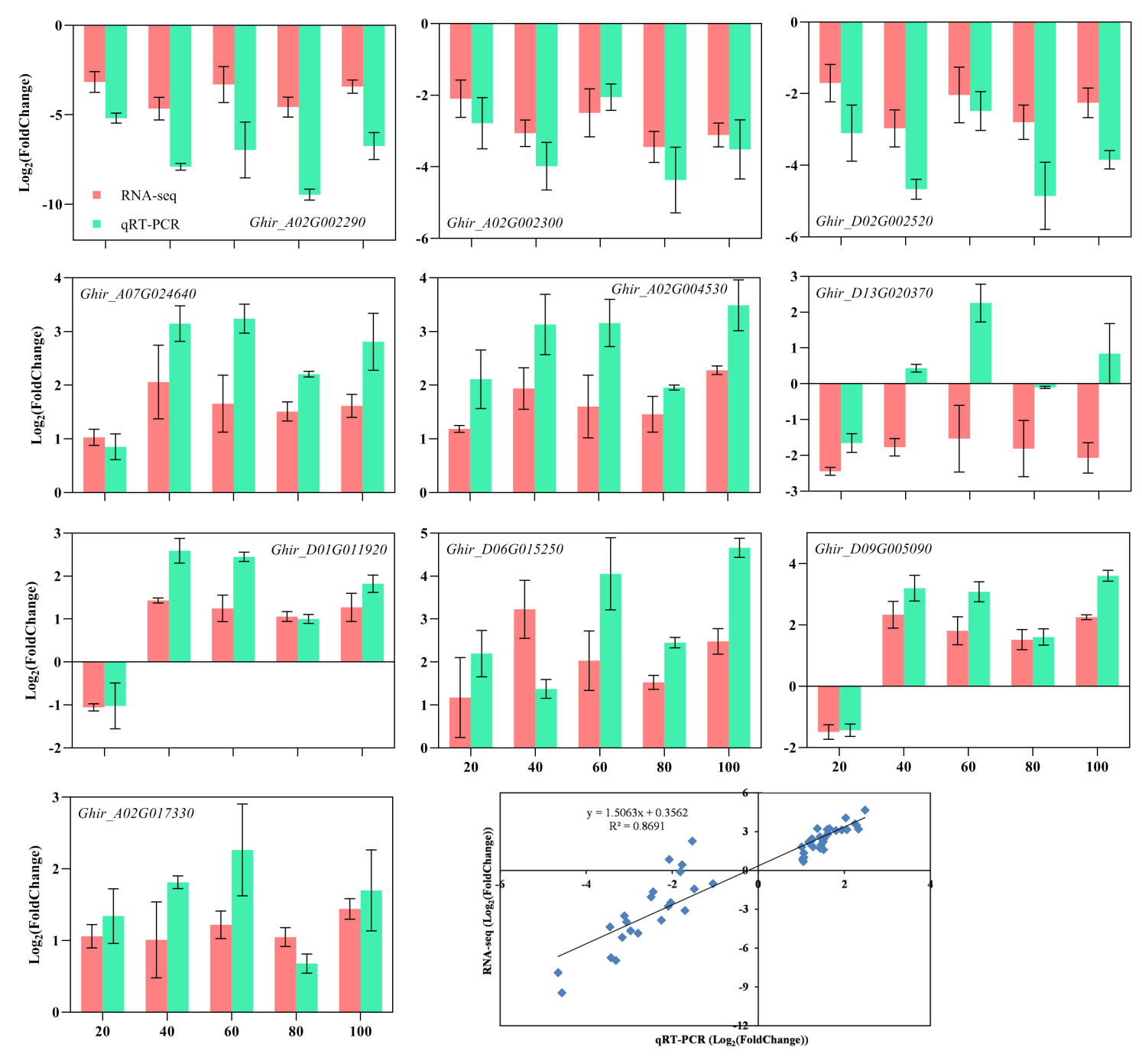
**

**FIGURE S9** Validation of RNA-Seq results using qRT-PCR.

## Supplementary Tables

**Table S1** Evaluation of the RNA-Seq data quality

| Sample  name | Raw  data (bp) | Clean  data (bp) | Clean bases  (G) | Error rate  (%) | Q20  (%) | Q30  (%) | GC  percent (%) |
| --- | --- | --- | --- | --- | --- | --- | --- |
| LN20_1 | 46706614 | 45572522 | 6.84 | 0.02 | 98.67 | 95.45 | 43.75 |
| LN20_2 | 41290668 | 39971960 | 6.00 | 0.02 | 98.61 | 95.32 | 43.83 |
| LN20_3 | 45323010 | 43924652 | 6.59 | 0.02 | 98.62 | 95.35 | 43.63 |
| LN40_1 | 45100328 | 43595360 | 6.54 | 0.02 | 98.67 | 95.44 | 43.45 |
| LN40_2 | 41037224 | 39897244 | 5.98 | 0.02 | 98.57 | 95.16 | 43.25 |
| LN40_3 | 47762152 | 46653490 | 7.00 | 0.02 | 98.65 | 95.43 | 43.4 |
| LN60_1 | 43062818 | 42004942 | 6.30 | 0.02 | 98.33 | 94.51 | 43.42 |
| LN60_2 | 45659974 | 44386664 | 6.66 | 0.02 | 98.59 | 95.23 | 43.27 |
| LN60_3 | 46281344 | 45084664 | 6.76 | 0.02 | 98.52 | 94.97 | 43.35 |
| LN80_1 | 44185546 | 43149450 | 6.47 | 0.02 | 98.56 | 95.13 | 43.29 |
| LN80_2 | 43205308 | 42273388 | 6.34 | 0.02 | 98.57 | 95.17 | 43.23 |
| LN80_3 | 45902754 | 44689044 | 6.70 | 0.02 | 98.58 | 95.21 | 43.29 |
| LN100_1 | 47319056 | 46328970 | 6.95 | 0.02 | 98.57 | 95.16 | 43.38 |
| LN100_2 | 47618084 | 46581038 | 6.99 | 0.02 | 98.71 | 95.54 | 43.06 |
| LN100_3 | 45648036 | 44574892 | 6.69 | 0.02 | 98.55 | 95.07 | 42.89 |
| NN20_1 | 46271852 | 44900940 | 6.74 | 0.02 | 98.64 | 95.39 | 43.57 |
| NN20_2 | 48251918 | 47191724 | 7.08 | 0.02 | 98.52 | 94.99 | 43.07 |
| NN20_3 | 52313574 | 51073062 | 7.66 | 0.02 | 98.48 | 94.93 | 43.28 |
| NN40_1 | 48457748 | 47475326 | 7.12 | 0.02 | 98.57 | 95.13 | 43.29 |
| NN40_2 | 44770788 | 43534756 | 6.53 | 0.02 | 98.59 | 95.23 | 43.28 |
| NN40_3 | 46837206 | 45733226 | 6.86 | 0.02 | 98.56 | 95.14 | 43.09 |
| NN60_1 | 46524038 | 45414530 | 6.81 | 0.02 | 98.57 | 95.14 | 43.22 |
| NN60_2 | 43933332 | 42392662 | 6.36 | 0.02 | 98.65 | 95.39 | 43.45 |
| NN60_3 | 44485534 | 43376834 | 6.51 | 0.02 | 98.60 | 95.25 | 43.43 |
| NN80_1 | 46208268 | 45119702 | 6.77 | 0.02 | 98.53 | 95.04 | 42.91 |
| NN80_2 | 46114580 | 44932544 | 6.74 | 0.02 | 98.57 | 95.25 | 43.31 |
| NN80_3 | 45254198 | 44179084 | 6.63 | 0.02 | 98.67 | 95.49 | 43.11 |
| NN100_1 | 46102494 | 44682658 | 6.70 | 0.02 | 98.64 | 95.42 | 43.59 |
| NN100_2 | 46999922 | 45776970 | 6.87 | 0.02 | 98.62 | 95.32 | 43.04 |
| NN100_3 | 47984112 | 46388266 | 6.96 | 0.02 | 98.73 | 95.6 | 43.24 |

**Table S2** The statistics of the comparison between reads and the conference genome

| Sample | Total reads  (bp) | Total map  (bp) | Total map rate  (%) | Unique map  (bp) | Unique map rate  (%) |
| --- | --- | --- | --- | --- | --- |
| LN20_1 | 45572522 | 44046178 | 96.65 | 42350546 | 92.93 |
| LN20_2 | 39971960 | 37757610 | 94.46 | 36174622 | 90.50 |
| LN20_3 | 43924652 | 40771954 | 92.82 | 39015000 | 88.82 |
| LN40_1 | 43595360 | 40519981 | 92.95 | 38740608 | 88.86 |
| LN40_2 | 39897244 | 36708124 | 92.01 | 35269134 | 88.40 |
| LN40_3 | 46653490 | 43819712 | 93.93 | 42148086 | 90.34 |
| LN60_1 | 42004942 | 39731711 | 94.59 | 38142834 | 90.81 |
| LN60_2 | 44386664 | 41518422 | 93.54 | 39789961 | 89.64 |
| LN60_3 | 45084664 | 42624073 | 94.54 | 40914605 | 90.75 |
| LN80_1 | 43149450 | 40654900 | 94.22 | 38966575 | 90.31 |
| LN80_2 | 42273388 | 38837598 | 91.87 | 37273714 | 88.17 |
| LN80_3 | 44689044 | 41994166 | 93.97 | 40311541 | 90.20 |
| LN100_1 | 46328970 | 39749426 | 85.80 | 38238444 | 82.54 |
| LN100_2 | 46581038 | 43161261 | 92.66 | 41479677 | 89.05 |
| LN100_3 | 44574892 | 40473059 | 90.80 | 38834846 | 87.12 |
| NN20_1 | 44900940 | 41309756 | 92.00 | 39656179 | 88.32 |
| NN20_2 | 47191724 | 43055932 | 91.24 | 41534520 | 88.01 |
| NN20_3 | 51073062 | 47175068 | 92.37 | 45352761 | 88.80 |
| NN40_1 | 47475326 | 43866364 | 92.40 | 42293547 | 89.09 |
| NN40_2 | 43534756 | 40148759 | 92.22 | 38481295 | 88.29 |
| NN40_3 | 45733226 | 41951749 | 91.73 | 40377264 | 88.29 |
| NN60_1 | 45414530 | 42606393 | 93.82 | 40999563 | 90.28 |
| NN60_2 | 42392662 | 40222979 | 94.88 | 38465814 | 90.74 |
| NN60_3 | 43376834 | 41453027 | 95.56 | 39820822 | 91.80 |
| NN80_1 | 45119702 | 41569375 | 92.13 | 39857611 | 88.34 |
| NN80_2 | 44932544 | 41918461 | 93.29 | 40196786 | 89.46 |
| NN80_3 | 44179084 | 40722581 | 92.18 | 39063151 | 88.42 |
| NN100_1 | 44682658 | 42620443 | 95.38 | 40842845 | 91.41 |
| NN100_2 | 45776970 | 42658877 | 93.19 | 40921610 | 89.39 |
| NN100_3 | 46388266 | 43806514 | 94.43 | 41921350 | 90.37 |

**Table S3** The primers for each reference gene for qRT-PCR analysis

| Gene name/Gene ID | Forward primer (5'-3') | Reverse primer (5'-3') |
| --- | --- | --- |
| *Actin-7-like*/LOC107911663 | GAACTGGAATGGTGAAGGC | TGACCCATCCCAACCATAAC |
| *NCED1*/Gh_A10G034400 | GGTGATCGGTTCTTGCAT | ATTCAGCCTGATTTCGGAC |
| *NCED6*/Gh_A10G034400 | CAACCCTCACTTCACTTCTT | GCGGGATTGACGATAAACT |
| *CYP707A2*/Gh_D03G138900 | ACAAGAAGGTGTTCGACG | GTCTCTTGAATCACCCTGG |
| *CYP707A4*/Gh_A06G007600 | ACGAAGTGAAAGATACGGGA | GCTGATTCGGTGCTCTTTAC |
| *PHT1-5*/Ghir_A02G002290 | ACAACACAGGTTTCCTCATC | CACAATGAATGTGGTGGAGT |
| *AMT1-1*/Ghir_A02G004530 | CATTCGGGTAGATCGGTG | CCAACCGAACCAAAGCAT |
| *NPF4.3*/Ghir_D06G015250 | TGCAGCAATCCGCAATAG | CAAAGCAGCTTCCTTGTCT |
| *HSF24*/Ghir_A02G017330 | ACGAGAACGAGAAGCTGA | GACCAGTTCGTCGCATTG |
| *PER55*/Ghir_A07G024640 | CAAGTGCTTAACACCGATCC | ACCCTTCCAAGTTTCCTCA |
| *RD21A*/Ghir_D01G011920 | TTTCCAAGGCCAGTGACC | ATCTTTGACTTCAGCGACAG |
| *CYP736*/Ghir_D09G005090 | AAGAATGGTGGAGGAAACTG | AAGGTGCAATAGGATGAAGC |
| *PHT1-5*/Ghir_D02G002520 | ATGGGTTTCTTCACTGACG | TCCAGGCTTTGCAGAATG |
| *PHT1-5*/Ghir_A02G002300 | TGCACTTCCAGCTCTTCTCA | CTGTTTGGCATTTCGGGCTA |
| *LAP1*/Ghir_D13G020370 | GTGCTATCAATGCAGCTCT | GCAACATGGTTTGACCGT |

**Table S3** The key genes that be identified

| Gene type | Gene ID |
| --- | --- |
| Phytohormone | *Ghir_A12G010870/Ghir_D12G015490/Ghir_D05G014030/*  *Ghir_D06G008630/Ghir_D02G023940/Ghir_D05G003720/*  *Ghir_D05G015750/Ghir_D13G024280/Ghir_A12G027630/*  *Ghir_D06G009100/Ghir_D07G001260/Ghir_D11G027410* |
| ROS detoxification system | *Ghir_D07G021980/Ghir_A02G002860/Ghir_A07G021890/*  *Ghir_D13G010870/Ghir_A09G017000/Ghir_D09G016460/*  *Ghir_D10G015610/Ghir_D11G014400/Ghir_D04G009280/*  *Ghir_D06G007460/Ghir_A06G007270/Ghir_A07G023600/*  *Ghir_D09G003210/Ghir_D12G007600/Ghir_A04G007750/*  *Ghir_A08G024060* |
| Cell cycle pathway | *Ghir_A08G021980/Ghir_A01G016710/Ghir_A06G004940/*  *Ghir_A06G013530/Ghir_D06G013730/Ghir_A13G004540/*  *Ghir_A03G020490/Ghir_D05G024100/Ghir_D11G036790/*  *Ghir_D09G011240/Ghir_D02G021370/Ghir_D11G002890/*  *Ghir_A11G002880/Ghir_D08G005160/Ghir_A08G006980/*  *Ghir_D06G020150* |
